# Genome-wide association study leads to novel genetic insights into resistance to *Aspergillus flavus* in maize kernels

**DOI:** 10.1186/s12870-020-02404-5

**Published:** 2020-05-11

**Authors:** Guomin Han, Cuiping Li, Fangzhi Xiang, Qianqian Zhao, Yang Zhao, Ronghao Cai, Beijiu Cheng, Xuewen Wang, Fang Tao

**Affiliations:** 1 School of Life Sciences, Anhui Agricultural University, Hefei, 230036 China; 2 National Engineering Laboratory of Crop Stress Resistance Breeding, Anhui Agricultural University, Hefei, 230036 China; 3 Department of Genetics, University of Georgia, Athens, 30602 USA

**Keywords:** Maize, *Aspergillus* pathogen, GWAS, SNP, Aflatoxin B1, Resistance

## Abstract

**Background:**

Fungus infection in staple grains affects the food storage and threatens food security. The *Aspergillus flavus* is known to infect multiple grains and produce mycotoxin Aflatoxin B1, which is mutagenic, teratogenic and causes immunosuppression in animals. However, the molecular mechanism of maize resistance to *A. flavus* is largely unknown.

**Results:**

Here we used corn kernels to investigate resistance genes to *A. flavus* using genome-wide association study (GWAS) of 313 inbred lines. We characterized the resistance levels of kernels after inoculating with *A. flavus*. The GWAS with 558,529 SNPs identified four associated loci involving 29 candidate genes that were linked to seed development, resistance or infection, and involved in signal pathways, seed development, germination, dormancy, epigenetic modification, and antimicrobial activity. In addition, a few candidate genes were also associated with several G-protein signaling and phytohormones that might involve in synergistic work conferring different resistance during seed development. Expression of 16 genes out of 29 during kernel development was also associated with resistance levels.

**Conclusions:**

We characterized the resistance levels of 313 maize kernels after inoculating with *A. flavus*, and found four associated loci and 16 candidate maize genes. The expressed 16 genes involved in kernel structure and kernel composition most likely contribute to mature maize kernels’ resistance to *A. flavus*, and in particular, in the development of pericarp. The linked candidate genes could be experimentally transformed to validate and manipulate fungal resistance. Thus this result adds value to maize kernels in breeding programs.

## Background

Corn, also known as maize (*Zea mays* L.), is one of the most important cereal crops, grown throughout the world for both feed stock and food. Food security has been frequently threatened by toxic contamination produced by fungal infection [1]. Fungal species in *Aspergillus* genus, e.g.*,* soil borne *A. flavus,* are known to produce aflatoxins, a carcinogenic mycotoxin affecting human and animals [1]. Corn and other grains are susceptible to *A. flavus* infection at pre- and post-harvest, leading to accumulation of toxic aflatoxin B1 which is very stable and difficult to remove during processing [2]. To prevent corn kernels from *A. flavus* infection, continuous efforts have been made to reduce infection throughout the past 50 years. It is well known that some corn accessions, e.g.*,* the Tuxpan in Mexico, possess high resistance to *A. flavus* and aflatoxin accumulation; therefore, incorporating high genetic resistance via breeding has been recognized as a promising solution. Several inbred lines with improved resistance to *A. flavus* through breeding selection have been reported by USDA-ARS and summarized in reviews [3–5]. Thus, it is of theoretical and applied importance to identify heritable genetic variants in maize conferring high resistance to *A. flavus* infection [3, 6].

Genes in plant-pathogen defense such as resistance (R) genes and signaling cascade genes should contribute to pathogen resistance in maize. However, resistance is usually regulated by multiple genes and interaction between maize and *A. flavus*, which results in difficulty in finding key genes. Using linkage mapping, quantitative trait loci (QTLs) were found on multiple chromosomes, e.g.*,* 1, 2, 3, 4, 5, 9 in crossing populations derived from high resistance line Mp313E and susceptible line Va35 or B73 [7, 8]. Another study, using a genome-wide association study (GWAS) showed a significant locus at chromosome 8 with QTL in recombination inbred lines derived from parent line RA and M53 [6]. Two QTLs in bins 6.06 centimorgan (cM) and 7.03 cM were believed to be the most promising for the marker-assisted introgression of the resistance present in Mp715 [9]. However, those reported QTL for resistance to *A. flavus* are still in large chromosomal regions, usually several centimorgans. A major QTL qAA8 region on chromosome 8 for *A. flavus* resistance was confirmed and refined by using 228 recombinant inbred lines (RILs) and an association population comprising 437 maize inbred lines [6]. No significant QTL associated with aflatoxin accumulation was observed in an association population of 346 maize inbred lines which were testcrossed to stiff-stalk line Tx714 [10]. By comparison, GWAS analysis identified 107 SNPs associated with aflatoxin accumulation by using an association mapping panel of 300 inbred lines which were crossed to a susceptible line Va35 [11]. Some genes exhibit higher expression levels within QTL bins in the highly resistant line MP313E than susceptible lines revealed by microarray expression analysis [12]. Genes in the jasmonic acid biosynthesis pathway may be associated with resistance to *A. flavus* according to GWAS analysis [13]. A survey of candidate genes for maize resistance to infection by *A. flavus* and/or aflatoxin contamination demonstrated that none of the genes identified in past studies or in-house studies is significant [14].

To date, the reference sequence of maize genome is available in several varieties [15–19]. The genetic variance, especially the SNPs and InDels, has been made public in recent years with the advances of high-throughput sequencing technology, like DNA re-sequencing [20, 21]. Li, et al. (2013) reported 556,944 SNPs in a panel of 368 maize accessions against maize B73 genome (version 2) [21]. Based on these DNA variants, several genotype-phenotype analyses have successfully discovered some gene loci via GWAS [22], e.g.*, ZmVPP1* encoding the pyrophosphatase which can improve drought tolerance [23], and *ZmFBL41* conferring banded leaf phenotype and sheath blight resistance in maize revealed by using a natural variation population [24].

In this study, we investigated the difference in resistance to *A. flavus* infection in post-harvest corn kernels in a 313-diversity panel. Combining with SNP variance, we aimed to identify genomic region associated with the resistance to *A. flavus* via GWAS analysis. Further functional screening of genes flanking 80 Kb region allowed us to identify gene candidates responsible for the resistance difference. Expression of 16 genes out of the 29 candidates during seed development was also checked for association with resistance levels. We successfully uncovered the genetic variance at four loci including several novel loci in maize conferring the resistance to *A. flavus* at post-harvest, which could guide genetic breeding improvement in maize for storage.

## Results

### Variations in kernel’s resistance to pathogen *A. flavus*

**Fig. 1:**
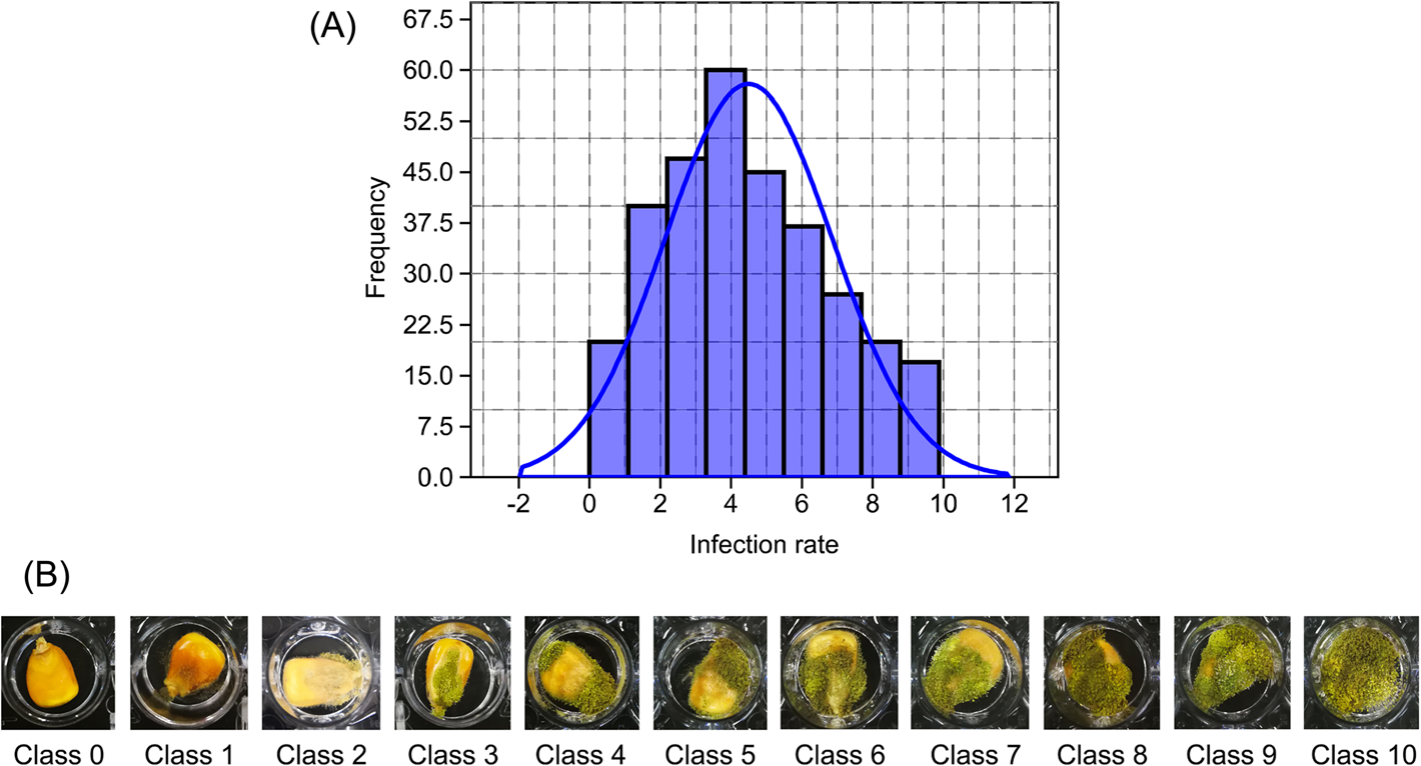
Infection rate of *A. flavus* in maize kernels. (**a**) The image shows a histogram of inoculated infection rate of *A. flavus* on the surface of maize kernels of 313 inbred lines in a germplasm pool. The curve shows the normal distribution. (**b**) The images of infection rate at class 0 to 10

**Fig. 2:**
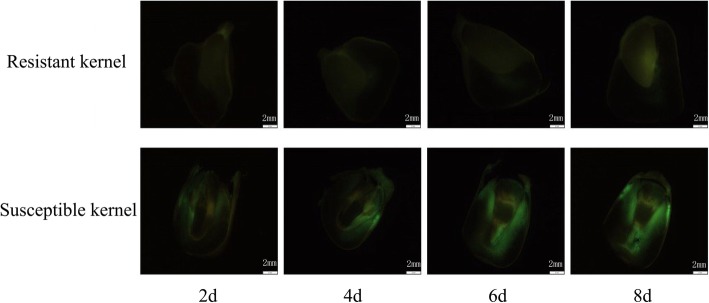
Comparison the penetration processes of *A. flavus* hyphae into different maize kernels. 2d, 2 days after inoculation with *A. flavus* conidia; 4d, 4 days after inoculation with *A. flavus* conidia; 6d, 6 days after inoculation with *A. flavus* conidia; 8d, 8 days after inoculation with *A. flavus* conidia

To examine the resistance to pathogen *A. flavus* infection, ~ 24 intact maize kernels from each of a panel consisting of 313 inbred lines [20, 21] were inoculated with *A. flavus* in a growth chamber for seven days. The rate of resistance was classified into eleven levels by measuring the ratio of infected area to the total kernel surface area (Fig. 1, Table S1). Thus, a lower infection ratio should reflect a higher resistance. We observed the variation in resistance to *A. flavus*, which follows a normal distribution. Sixty inbred lines with the highest frequency had an infection rate around 4. Seventeen and 20 lines had a higher infection ratio (> 9) and lower infection rate (< 1), respectively (Fig. 1). The susceptible kernels had visible mycelia inside while the kernels of resistant inbred lines did not (Fig. 2). Additionally, less Aflatoxin B1 was produced in the kernels of resistant inbred lines than that of susceptible lines (Fig. S1).

### Genetic loci associated with resistance to *A. flavus* via GWAS

**Fig. 3:**
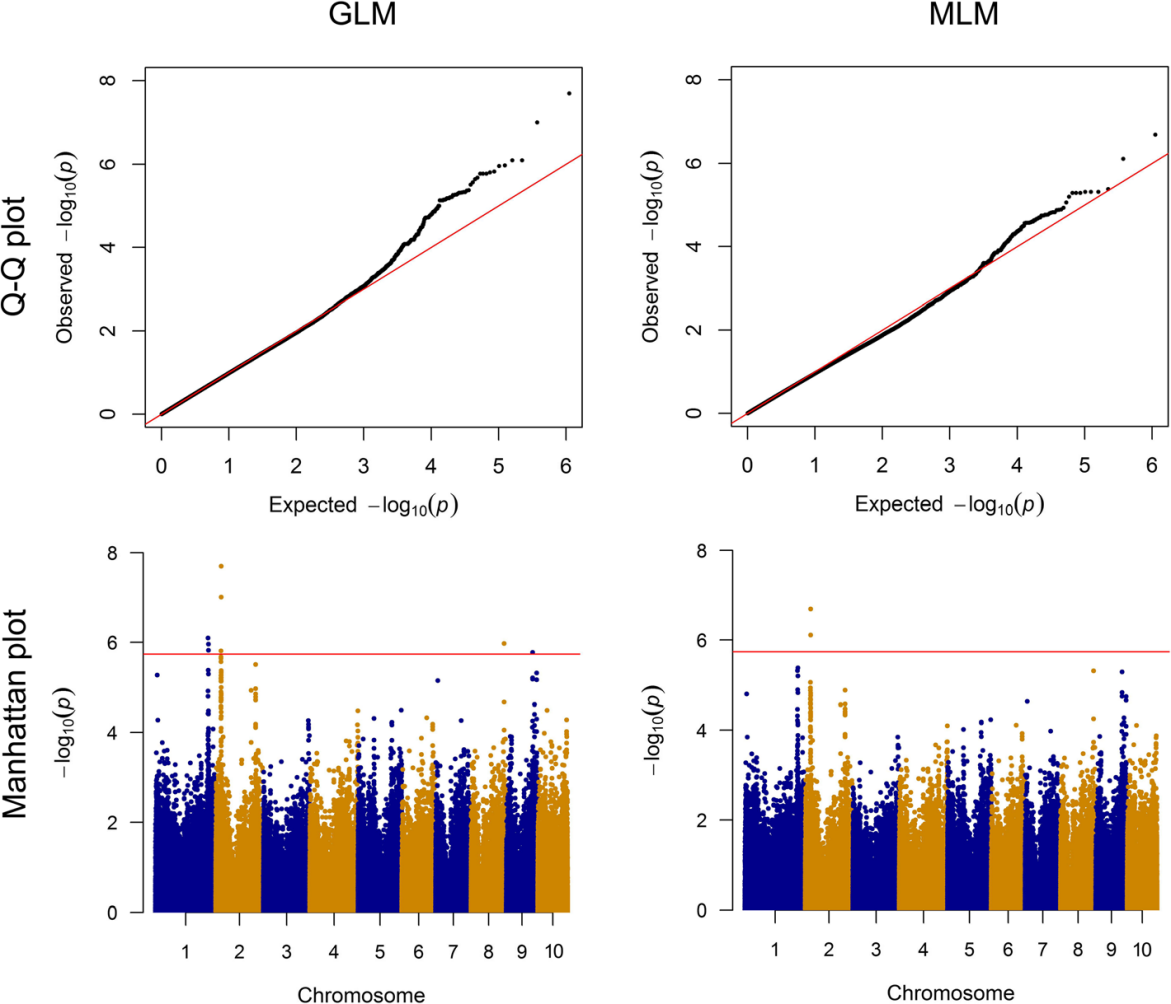
Q-Q plot and Manhattan plot showing associated loci for resistance to *A. flavus* on maize kernels via the general linear model (GLM) and mixed linear model (MLM). The SNP locus was plotted in dots and the loci above the red line are considered to be significant at threshold of -log10(p) > 1.8 × 10^− 6^

To investigate the associated gene candidates conferring the resistance to *A. flavus* at whole genome scale, we carried out a SNP-based GWAS. After filtration of minor allele frequency (MAF) < 0.05, a total of 556,790 SNPs were used to identify the associated loci via the general linear model (GLM) and mixed linear model (MLM) [21, 25]. Association analysis via GLM identified four SNP regions significantly associated with resistance at the threshold of *p* ≤ 1.8 × 10^− 6^, distributed throughout four chromosomes (Fig. 3). The GWAS analysis detected that four, three, one, and three SNPs were associated with regions on chromosome 1, 2, 8 and 9, respectively (Table 1). Compared with GLM, only one SNP region was detected on chromosome 2 via MLM. Two associated SNPs on chromosome 2 were the same in regard to the SNPs that were identified by GLM (Table 1). Fewer associated SNPs were detected via MLM than that of GLM (Fig. 3). Here, the results of GLM were used for subsequent analyses.

**Table 1:**
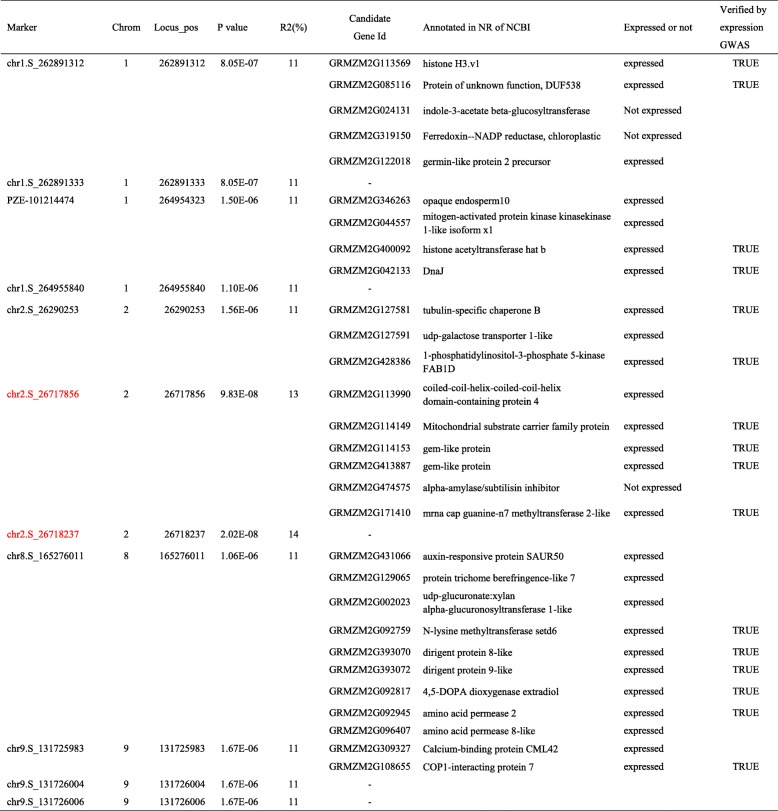
Genetic loci and candidate genes associated with resistance to *A. flavus* identified via GWAS

Note: The black SNP markers, SNPs detected only using GLM; the red markers, SNPs detected using GLM and MLM

### Candidate genes in maize confer resistance to pathogen *A. flavus*

**Fig. 4:**
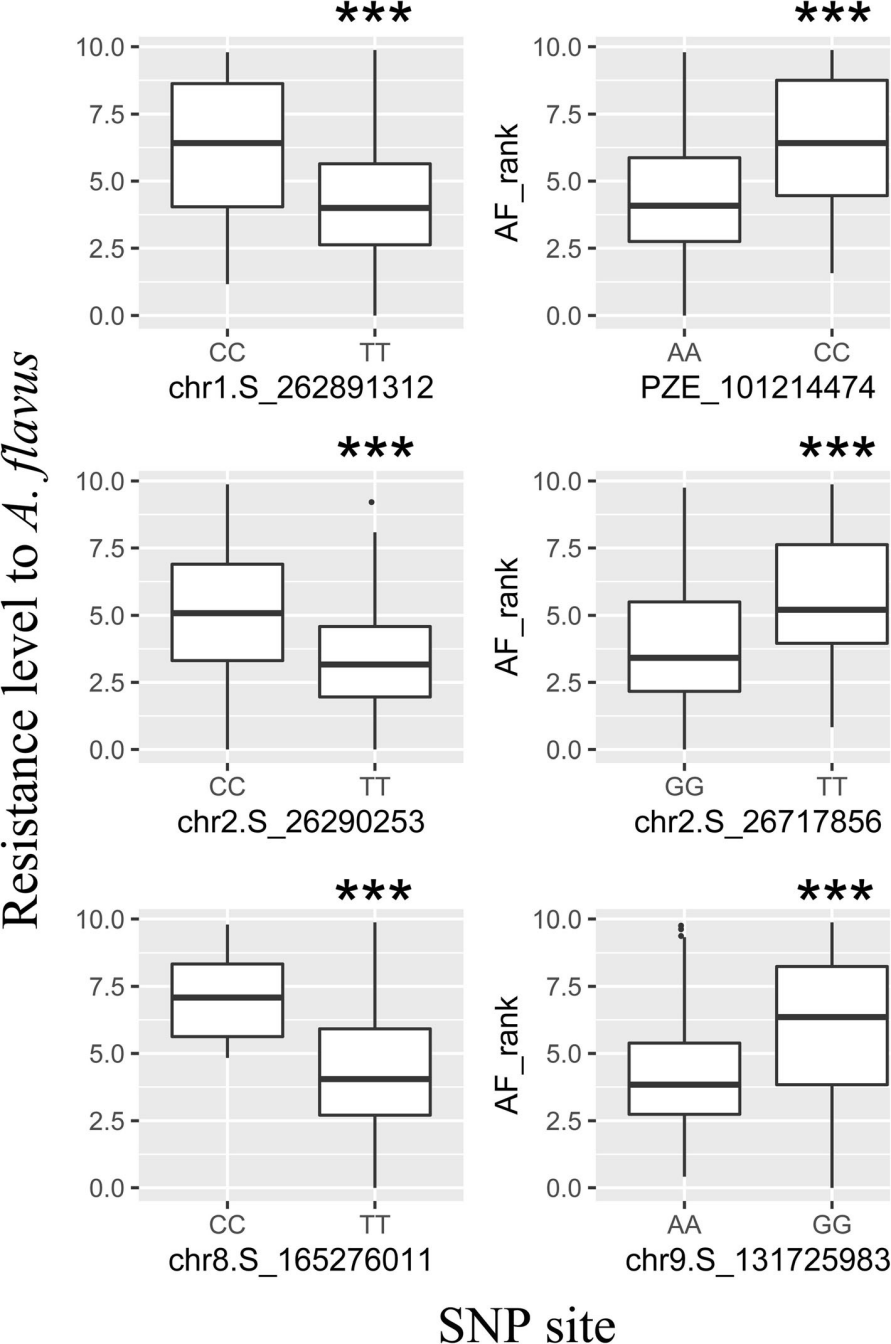
Boxplot of resistance rank to *A. flavus* at significantly associated SNPs. The resistance levels of all inbred lines at vertical axis were box-plotted along the SNP alleles at the horizontal axis, *** indicates *p* < 0.001 via T-test

The SNP loci identified by GWAS provide clues for understanding the genetic architecture which confers the variation in resistance. The maize inbred lines and SNP data have been used to investigate oil biosynthesis, drought tolerance, banded leaf and sheath blight resistance, etc., and 50 kb to 100 kb region of the peak SNP were often used to search candidate genes [21–24]. To resolve candidate genes responsible for *A. flavus* resistance, we further extracted the genes flanking 80 kb at either side of the associated SNP locus and within the significant associated region, and then annotated their functions against databases to identify the most likely gene candidates. After comparing the annotation of genes in maize genome assembly between version V2 and V4 (Table S2), we kept the consistent genes and the genes with additional evidence as the final gene set. Nine, nine, nine and two candidate genes were associated with two, two, one, and one SNP region on chromosome 1, 2, 8 and 9, respectively (Table 1). Further boxplot analyses showed that the polymorphisms of all six SNPs fit well with the variation of each resistance ranks (Fig. 4), which suggested that the six SNPs are likely associated with genes conferring the resistance to *A. flavus.*

### Relation of gene expression and resistance to *A. flavus* in the germplasm panel

**Fig. 5:**
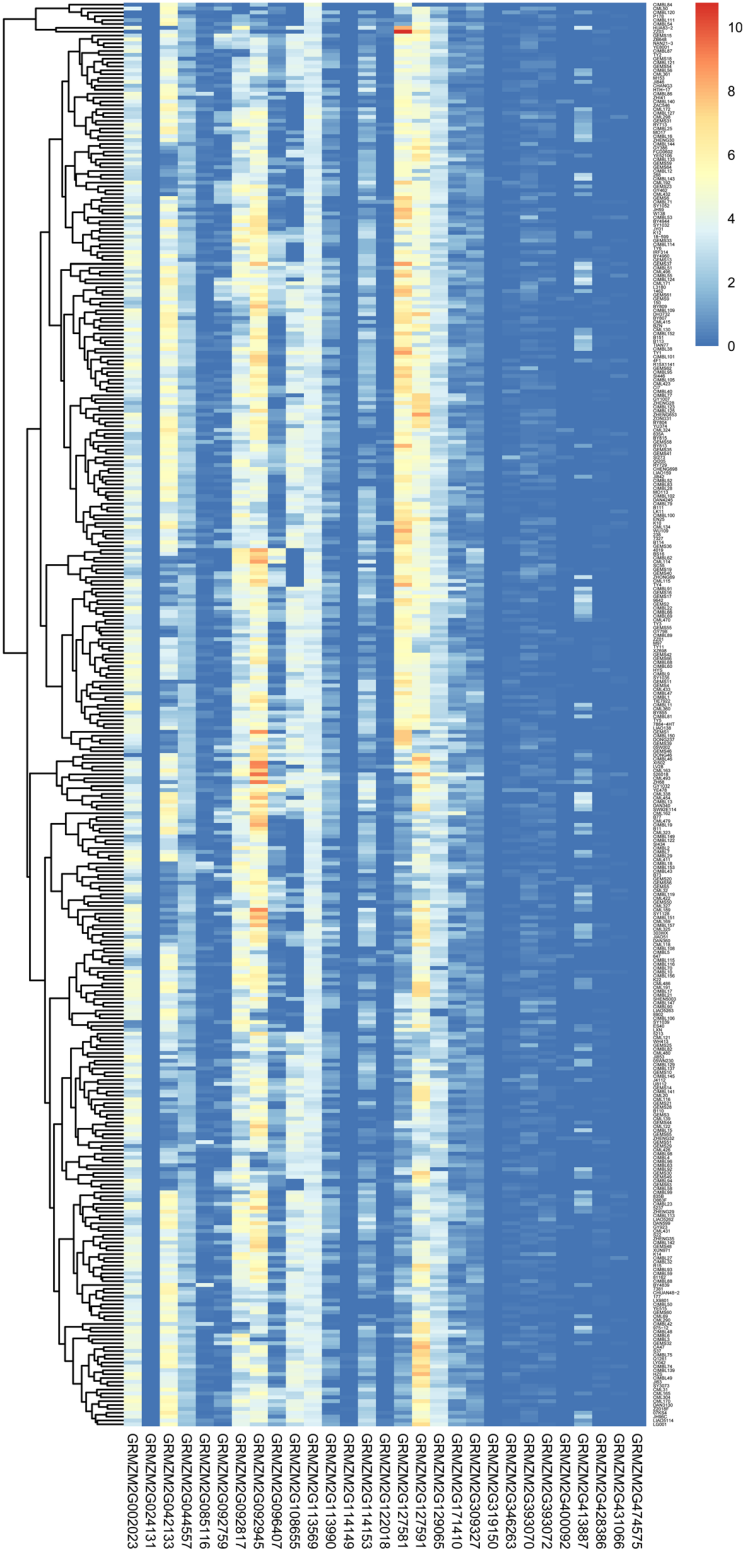
The expression levels of the candidate genes in 368 inbred lines. The expression data are from seed tissue at 15 days old after pollination in the study of Fu et al. (2013)

To indentify the expression of each candidate gene, raw transcriptome profiles in the developmental maize kernel of 368 inbred lines at 15 days after pollination (DAP) were downloaded from NCBI and analyzed with the HISAT2 tool. Results show that the majority of resistance associated genes were expressed during the development of kernel in inbred lines with the exception of genes GRMZM2G024131, GRMZM2G319150, and GRMZM2G474575 which were not, or were very lowly expressed, in all inbred lines (Fig. 5 and Table S3). The abundance of transcripts expressed from these genes was used to conduct a SNP-expression association analysis via GWAS [20]. Consistent with resistance-SNP association results, sixteen out of 29 genes were associated with the flanking 80 kb region (Table 1), indicating that the sixteen expressed genes involving in kernel structure and kernel composition are most likely contributing to mature maize kernels’ resistance to *A. flavus*.

**Fig. 6:**
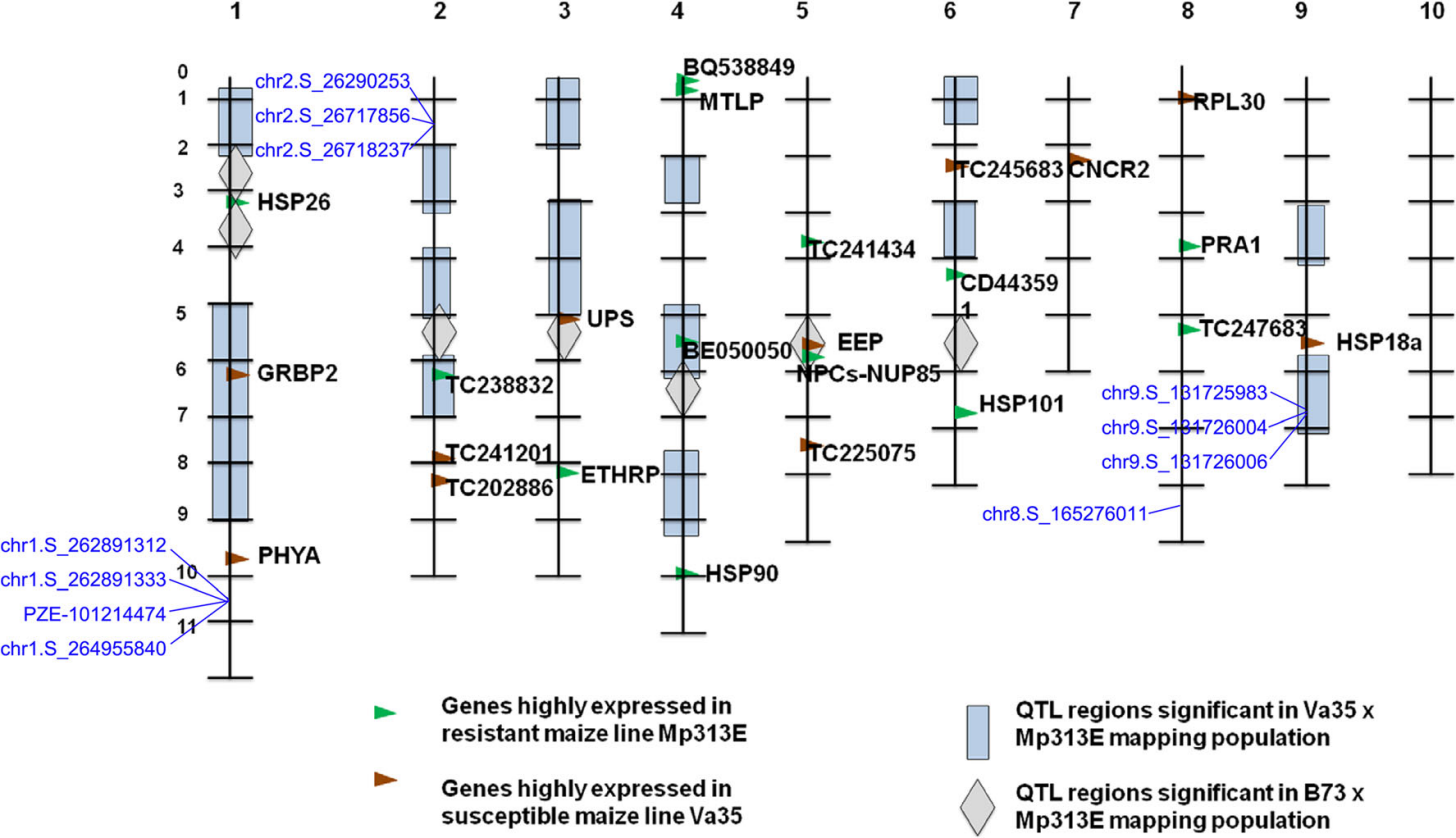
Comparison the SNP loci in this study with loci reported previously. The previously reported loci are from Kelley, et al. (2012). The SNP loci in this study were indicated as blue color

It is worth noting that three adjacent SNPs (chr9.S_131,725,983, chr9.S_131,726,004, and chr9.S_131,726,006) on chromosome 9 in this study overlapped with a loci previously identified in pre-harvest resistance to *A. flavus* in a study conducted by Kelley, et al (2012) (Fig. 6) [12], indicating a common genomic feature for resistance. In this region, gene GRMZM2G309327 and GRMZM2G108655 are the most important candidate genes in pre-harvest investigations. The gene GRMZM2G309327, as a homolog to AT4G20780 and AT5G44460 in Arabidopsis, encodes Calcium-binding protein (CML42) which is known to function in signal transportation during infective defense in plant [26, 27]. This gene is also expressed in the pericarp/aleurone of the corn kernel [28]. The gene GRMZM2G108655 encodes COP1-interacting protein 7 involving in anthocyanin biosynthesis [29], indicating that the gene can influence seed coloration.

Besides the three adjacent SNPs on chromosome 9, the remaining associated SNPs are newly identified in the association results to *A. flavus* resistance. Many candidate genes were reported to be involved in seed development. The associated loci on Chr2 is close to previously reported QTL loci resistant to *A. flavus* [7]. At the loci (Table 1), gene GRMZM2G127591 is homologous to *Arabidopsis* AT1G21870, encoding GOLGI NUCLEOTIDE SUGAR TRANSPORTER 5, which has been reported to be required for synthesis of essential cell surface component [30]. Among the genes at loci on Chr8, gene GRMZM2G129065 encodes a TRICHOME BIREFRINGENCE-LIKE protein, which is homologous to Arabidopsis gene AT1G48880. This gene is involved in the synthesis and deposition of secondary wall cellulose by influencing the esterification state of pectic polymers [31, 32]. The gene GRMZM2G400092 encodes a histone acetyltransferase (HAT) B which is involved in acetylation of newly synthesized histone H4 during embryo germination. HAT B is also involved in seed development and germination in maize [33, 34]. GRMZM2G393070 and GRMZM2G393072 are dirigent proteins which were reported to mediate lignan and cyanogenic glucoside formation in Flax seed [35].

## Discussion

The seed coat of flowering plants plays important roles in seed germination, vigor and longevity potentials [36]. It is also the first surface of seeds’ defense against adverse external factors [37]. The seed coat of maize kernel contains two outer layers of the kernel, called the pericarp and the aleurone. Approximately 87% of the pericarp is crude fiber which is mainly constituted of hemicellulose (67%), cellulose (23%) and lignin (0.1%) [38]. The tough fiber of pericarp forms a shield to protect the interior of seed from abiotic and biotic stresses including infection of mold and bacteria [36, 39]. Once *A. flavus* penetrates the seed coat or pericarp of kernels, the fungal mycelia can grow inside the kernel of susceptible inbred lines (Fig. 2). Generally, the more the fungi penetrated into the maize kernels, the higher Aflatoxin B1 was detected in kernels post 7 days of inoculation with *A. flavus* conidia (Fig. S1). The results indicated that the intactness of maize seeds should play a vital role in resistance to *A. flavus* infection in post-harvest corn kernels.

The seed coat also acts as channel for transmitting environmental cues, such as water into the interior of the seed in addition to as a shield [37]. After absorbing water, maize seeds will swell. The integrity of the pericarp should play a vital role in the kernel resistance to mold, and the rate of kernel resistance to *A. flavus* reflects the integrity of seed after assimilation of water. The diversity of the resistance levels in this study indicated that the integrity of seeds of inbred lines should be different after absorbing water. Among the inbred lines, results of 17 seed lines with a higher infection ratio (> 9) while 20 seed lines with a lower infection rate (< 1) suggest that the lines with lower infection rate (< 1) were more intact than that of lines with a higher infection ratio (> 9) during the fungal infection process. In this study, two, two, one, and one SNPs with 4, 6, 5 and 1 candidate genes, which were further verified by SNP-expression GWAS analysis, were associated with regions on chromosome 1, 2, 8 and 9, respectively. The six SNPs could explain 11 ~ 14% of the phenotypic variation. The majority of genetic loci associated with resistance to *A. flavus* in this study were different from the QTL regions in the study of Kelley, et al (2012) [12], which might be due to the fact that different development stages and storage condition of seeds were used. Fourteen-day-old seeds after self-pollination were inoculated with the fungus *A. flavus* strain NRRL 3357 in their study [12] while dry mature seeds were used in our study, which should contribute to the difference.

The intactness of maize seeds after absorbing little water might be controlled by the integrity of seed coat and the moisture absorption capacity of the endosperm. Seeds with cracking or broken seed coats are prone to mold infection [37, 40, 41]. A mature seed is developed from the zygote which is double fertilized, resulting in the formation of a diploid embryo and a triploid endosperm [37, 42]. In contrast to the embryo and endosperm, the seed coat is entirely maternal in origin [37]. To understand kernel development, immature seeds of 368 inbred lines at 15 DAP were investigated by RNA sequencing [20]. The gene expression profile of the lines is highly variable, and relationships between expression quantitative trait loci (eQTL) and their targets were established to reveal gene regulatory networks [20]. Although the expression levels of the candidate genes in maize kernels without *A. flavus* inoculation should not be directly involved in fungal resistance during kernel development, at least some of the expressed genes could be the candidate genes contributing to the different components of kernels. It has been summarized that seed size is determined by several pathways including the IKU pathway, the ubiquitin-proteasome pathway, G-protein signaling, the mitogen-activated protein kinase signaling pathway, phytohormones and transcriptional regulatory factors [42]. The pathways can influence the formation of seed size by regulating the endosperm and/or maternal tissue growth. In this study, some candidate genes belong to G-protein signaling pathways (GRMZM2G113569, and GRMZM2G309327), while other genes are related to phytohormones (GRMZM2G431066, GRMZM2G114153, GRMZM2G413887 and GRMZM2G092759). The genes involved in the development of seed size were also the associated genes which contributed to *A. flavus* resistance*.* In addition, several associated genes were reported to be involved in the synthesis of the embryo, the endosperm, and seed coat. For example, GRMZM2G127591 and GRMZM2G092945 are engaged in the transportation of sugars and amino acids during seed development [30, 43]. GRMZM2G129065, GRMZM2G393070 and GRMZM2G393072 might be involved in the syntheses of polymers, e.g., pectin, lignin [31, 32, 35]. The G-protein signaling and phytohormones related genes and other candidate genes should synergistically work for the seed development, resulting in mature seeds with different capacity against adverse environmental conditions. Some seeds of the inbred lines will be of high resistance to mold infection, including *A. flavus.* The validation and further characterization of the detailed roles of likely gene candidates during the response to *A. flavus* infection could be of high priority in future experiments, such as gene overexpression and gene knock-out analysis.

## Conclusions

We characterized the resistance levels of 313 maize kernels after inoculating with *A. flavus*. Four loci and 16 candidate maize genes were mined via GWAS. The 16 expressed genes involving in kernel structure and kernel composition most likely contributed to resistance of mature maize kernels to *A. flavus*, particularly, genes involving in the regulating development of pericarp. The linked candidate genes could be experimentally transformed to validate and manipulate resistance to add value to maize kernels in breeding programs.

## Methods

### Plant materials

Three hundred and thirteen maize inbred lines, including tropical/subtropical inbred lines, temperate lines and lines of mixed origin, were kindly provided by Prof. Guoying Wang (Institute of Crop Science, Chinese Academy of Agricultural Sciences). All the inbred lines were planted in the field of Sanya (Hainan Province, 18.75 N, 109.17E) in November 2015. The mature seeds of each inbred line were used for fungal infection analysis.

### Analysis of maize resistance to fungal infection

A laboratory kernel-screening assay (KSA) was performed as described by Brown et al. (1999) with modifications [44]. Briefly, the surface of ~ 24 kernels from each inbred line was sterilized with 75% ethanol and 1% NaClO for 5 min and dipped into a suspension of *A. flavus* conidia (4 × 10^6^ cfu/mL) for 5 min. Kernels were then placed in a 24-well plate individually, one kernel in each well, and incubated at 28 °C for 7 days. High humidity (> 95% RH) was maintained by supplying sterile water to the space between holes. Two biological replicate experiments were conducted for each inbred line. Infection was designed as visible mycelial and conidia on the surface of the kernel, and the infection was classified by the ratio of the infected area to the surface area of each kernel. Eleven classes were defined as follows: 0 = no visible mycelial and conidia, 1 = 1–10%, 2 = 21–30%, 3 = 31–40%, 4 = 41–50%, 5 = 51–60%, 6 = 61–70%, 7 = 70–80%, 8 = 80–90%, 9 = 90–100%, 10 = 100%.

Aflatoxin B1 was extracted and the abundance of Aflatoxin B1 was determined by HPLC with a fluorescence detector [2]. The kernels were dried for two days in a 60 °C oven and ground into powder. The Aflatoxin B1 were extracted in methanol: H_2_O = 70:30 (v/v), shaking at 190 rpm at 20 °C for two hours, followed by centrifuging for five mins at 8000 rpm. The supernatant was filtered through 0.22 μm filter and filter-through liquid was used for HPLC analysis.

### Genome-wide association study and annotation of candidate genes

558,529 SNPs, population structure and kinship data were kindly provided by Prof. Guoying Wang and Prof. Jianbing Yan (http://www.maizego.org) [20, 21]. 558,529 SNPs in the maize germplasm pool were used to carry out GWAS with TASSEL (v5.0) [21, 25]. General Linear Model (GLM) and Mixed Linear Model (MLM) were used to identify the genetic loci associated with resistance to *A. flavus* infection as described previously [23]. To balance false negative and false positive associations in GWAS, two workflows were used: GLM plus principal component analysis (PCA) for population structure inference and principle components 1 and 2 as covariates, and MLM plus PCA as well as kinship by using Tassel 5 workflows. In addition, we also used the previous generated population structure information and kinship data to conduct GWAS [20, 21]. Both input data sets yielded the same results. Manhattan and Q-Q plots were generated via the ‘qqman’ package in R. If the *P*-value was lower than Bonferroni-adjusted significance threshold (1.8 × 10^− 6^), the SNP was considered significant locus [21]. All the genes within 80 kb flanking region either upstream or downstream of the significant SNP site were further annotated via blastp against Nr of NCBI, InterProScan (swiss-prot), and Pfam [45, 46].

### Analysis of the expression level of candidate genes

The raw transcriptome profiles (BioProject: PRJNA208608) of maize kernels at 15 days after pollination were obtained from NCBI. The SRA format data were transformed into fastq format via NCBI fastq-dump in sratoolkit (version 2.9.2). The raw reads were trimmed to remove low quality base-calls and adaptor sequences using the Trimmomatic (v0.33) tool [47]. Cleaned reads were mapped to the genome of *Z. mays* B73 (V2) via HISAT2 (v2.1.0) [48]. StringTie was applied to assemble and integrate the transcripts in each sample [48]. The expression level of each gene was calculated by Ballgown [48]. The expression of each candidate gene was used to carry out GWAS again to search for SNP-expression association via TASSEL (v5.0) [25].

## Supplementary information

**Additional file 1 Fig. S1** The content of Aflatoxin B1 in kernel of some maize lines after 7 days of inoculation with *A. flavus.***Table S1** Resistance level of the inbred lines. **Table S2** Comparison of the protein IDs in V2 version with that in V4 version of maize B73. **Table S3** The expression levels of the candidate genes in 368 inbred lines

## Data Availability

The SNP data is available at http://www.maizego.org/Resources.html. The file name of the SNP data is ‘SNPs_368lines_maf0.05_0.56 M.hmp.gz’ at https://pan.baidu.com/s/1t_S_kof4S8G1bj_j4_c4bQ#list/path=%2FMaizeGo%20Resources%2FGenotype [20, 21]. All other data and material used in this study are available from the corresponding author upon reasonable request.

## Abbreviations

GWAS: Genome-wide association study
QTL: Quantitative trait loci
MAF: Minor allele frequency
ABA: Abscisic acid
HAT: Histone acetyltransferase
KSA: Kernel-screening assay
GLM: General Linear Model
MLM: Mixed Linear Model
