# Supplementary material for: Genome-wide association study leads to novel genetic insights into resistance to Aspergillus flavus in maize kernels
Source: BMC Plant Biol. 2020 May 11;20:206. doi: 10.1186/s12870-020-02404-5 (PMC7216483; doi:10.1186/s12870-020-02404-5)
Supplement: Supplementary file 1 — Additional file 1 Fig. S1 The content of Aflatoxin B1 in kernel of some maize lines after 7 days of inoculation with A. flavus.Table S1 Resistance level of the inbred lines. Table S2 Comparison of the protein IDs in V2 version with that in V4 version of maize B73. Table S3 The expression levels of the candidate genes in 368 inbred lines [file 12870_2020_2404_MOESM1_ESM.pdf]

Supplementary Figures and Tables

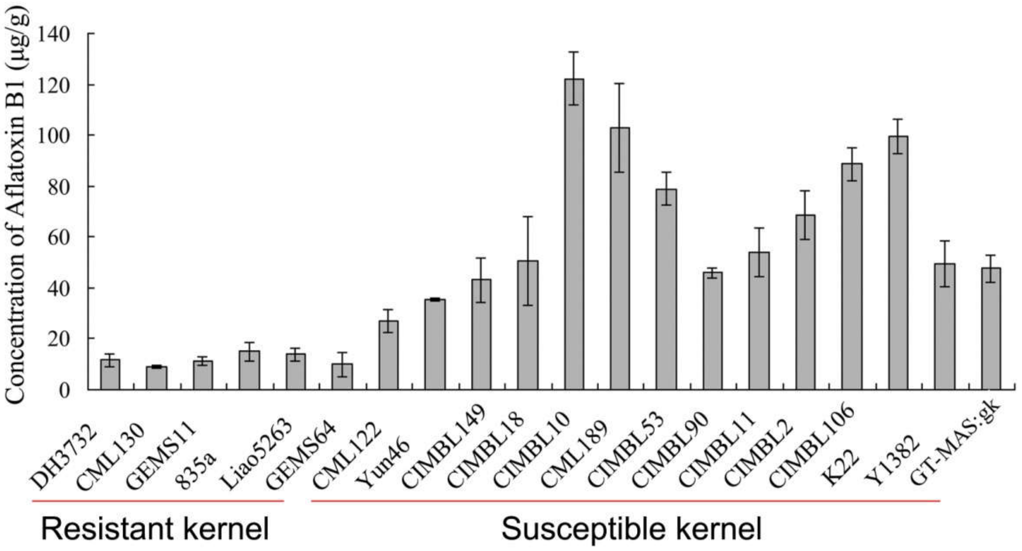

Figure S1 The content of Aflatoxin B1 in kernel of some maize lines after 7 days of inoculation with *A. flavus*

Table S1 Resistance level of the inbred lines

Inbred line	Resistance level	Sub populations
CIMBL25	0	TST
CIMBL86	0	TST
150	5.917	MIXED
177	3.083	NSS
238	1.958	NSS
268	3.25	NSS
501	1.542	NSS
1462	4.208	MIXED
4019	7.5	NSS
5213	4.333	MIXED
7381	5.042	NSS
8902	6.304	MIXED
9642	6.417	MIXED
9782	4.208	NSS
526018	6.542	NSS
04K5702	0.708	NSS
05W002	6.958	MIXED
05WN230	3.833	MIXED
07KS4	6.792	NSS
18-599	9.375	NSS
303WX	6.617	MIXED
335F	4.167	Mixed
335M	4.667	Mixed
384-2	7.083	Mixed
3H-2	3.625	Mixed
478-BC6	4.083	Mixed
4F1	1.042	NSS
7884-4Ht	2	Mixed
835a	1.875	NSS
835b	3.417	NSS
975-12	3.958	MIXED
B11	4.917	TST
B110	5.167	MIXED
B111	2.333	MIXED
B113	4.125	NSS
B151	3.792	MIXED
B73	5.208	SS
BGY	0.875	Mixed
By4944	7.708	NSS
By4960	6.708	NSS
By807	3.375	NSS
By809	5.667	NSS
By813	1.75	NSS
C8605-2	4.542	Unknown
CA47	4	TST
CF773-2	3.25	Mixed
Chang7-2	1.167	Mixed
Cheng698	7.417	Mixed
chuan48-2	3.042	Mixed

CIMBL1	2.083	NSS
CIMBL10	8.625	MIXED
CIMBL100	5.917	TST
CIMBL101	9.208	TST
CIMBL102	3.042	TST
CIMBL105	6.542	TST
CIMBL106	8.625	TST
CIMBL108	8.792	TST
CIMBL109	3.292	TST
CIMBL11	9.083	TST
CIMBL110	6.083	TST
CIMBL111	9.583	TST
CIMBL113	5.667	TST
CIMBL114	6.875	TST
CIMBL115	5.417	TST
CIMBL116	7.75	TST
CIMBL123	4.042	TST
CIMBL124	2.75	TST
CIMBL125	2.292	TST
CIMBL127	6.542	TST
CIMBL13	8.833	MIXED
CIMBL133	5.208	TST
CIMBL136	7.708	TST
CIMBL14	3.625	TST
CIMBL140	3.792	TST
CIMBL141	3.417	MIXED
CIMBL142	8	MIXED
CIMBL144	1.5	MIXED
CIMBL145	4.083	SS
CIMBL146	0.625	Mixed
CIMBL147	5.167	TST
CIMBL149	9.792	TST
CIMBL151	4.125	TST
CIMBL152	2.667	TST
CIMBL153	9.875	TST
CIMBL154	5.833	Mixed
CIMBL155	5.375	TST
CIMBL156	6.5	TST
CIMBL157	4.667	TST
CIMBL16	3	TST
CIMBL17	2.583	MIXED
CIMBL18	8.75	TST
CIMBL19	5.958	TST
CIMBL2	8.542	TST
CIMBL20	5.739	TST
CIMBL21	2.917	TST
CIMBL22	1.583	TST
CIMBL23	5.167	TST
CIMBL27	3.417	TST
CIMBL28	3.25	TST
CIMBL32	3.063	TST

CIMBL38	2.5	TST
CIMBL4	7.042	TST
CIMBL40	3.75	TST
CIMBL42	0.833	TST
CIMBL43	7.042	TST
CIMBL44	5.043	TST
CIMBL47	0.417	TST
CIMBL48	5.417	TST
CIMBL5	8.417	TST
CIMBL51	1.167	TST
CIMBL52	6.25	TST
CIMBL53	8.75	TST
CIMBL54	3.042	TST
CIMBL55	2.833	TST
CIMBL56	2.708	TST
CIMBL57	5	TST
CIMBL59	3.667	TST
CIMBL62	2.917	TST
CIMBL66	3.083	TST
CIMBL67	0.917	TST
CIMBL68	3.375	TST
CIMBL69	1.875	TST
CIMBL7	3.958	TST
CIMBL70	7.833	TST
CIMBL71	4.792	TST
CIMBL73	3.833	TST
CIMBL74	2.042	TST
CIMBL75	4.667	TST
CIMBL77	5.417	TST
CIMBL8	1.625	TST
CIMBL81	1.125	TST
CIMBL82	4.083	TST
CIMBL83	3.75	TST
CIMBL84	3.125	TST
CIMBL87	1.458	TST
CIMBL89	0.708	TST
CIMBL9	3.25	MIXED
CIMBL90	9.75	TST
CIMBL92	5.833	TST
CIMBL93	0.545	TST
CIMBL95	5.5	MIXED
CIMBL96	4.458	TST
CIMBL97	7.458	TST
CIMBL98	3.833	TST
CIMBL99	6.917	TST
CML114	3.208	TST
CML115	5.042	TST
CML116	2.875	TST
CML118	4.042	TST
CML121	3.375	TST
CML122	8.375	TST

CML130	1.708	TST
CML134	4.458	TST
CML169	7.833	TST
CML170	1.174	TST
CML171	6	TST
CML189	8.292	TST
CML192	2.167	TST
CML20	6.417	TST
CML223	3.25	TST
CML225	7.042	TST
CML290	5.292	TST
CML298	0.75	TST
CML300	3.083	TST
CML304	5.083	TST
CML31	6.333	TST
CML32	5.75	TST
CML323	4.167	TST
CML324	7.458	TST
CML325	4.708	TST
CML327	6.375	TST
CML338	4.833	TST
CML361	9.375	TST
CML364	6.667	TST
CML411	9.583	TST
CML415	3.375	TST
CML423	2	TST
CML430	2.215	TST
CML433	0.667	TST
CML454	1.458	TST
CML479	3.667	TST
CML486	7.083	TST
CML493	3.542	TST
CML50	0.417	TST
CML51	4.875	TST
CML69	7.5	TST
D863F	6.375	NSS
Dan3130	2.708	NSS
Dan340	5.292	NSS
Dan360	7.833	NSS
Dan4245	6.333	Mixed
Dan598	4.583	NSS
Dan599	2.792	NSS
DE.EX	2.25	NSS
DH3732	1.083	NSS
DL1	3.458	Unknown
EN25	4.25	MIXED
ES40	3.167	MIXED
FCD0602	1.917	MIXED
GEMS11	0.875	SS
GEMS14	1.917	SS
GEMS16	4.583	SS

GEMS18	3.167	SS
GEMS19	5.083	MIXED
GEMS2	7.25	MIXED
GEMS20	4.708	SS
GEMS21	5.917	SS
GEMS23	0.667	SS
GEMS25	6.333	MIXED
GEMS28	3.125	MIXED
GEMS30	2.875	MIXED
GEMS31	5	TST
GEMS33	2.5	NSS
GEMS39	3.708	NSS
GEMS4	4.167	SS
GEMS41	5.625	NSS
GEMS42	9.625	NSS
GEMS51	5.167	SS
GEMS53	1.75	SS
GEMS55	3.125	SS
GEMS59	6.583	NSS
GEMS6	5.875	SS
GEMS60	5.708	NSS
GEMS61	2.083	SS
GEMS63	8.5	SS
GEMS64	0.708	SS
GEMS65	5.583	SS
GEMS9	1.25	SS
GL178	3.875	Unknown
Gy220	3.833	NSS
Gy237	6.292	NSS
Gy386	3.583	NSS
Gy462	3.5	NSS
Gy923	6.625	NSS
HZ4	6.958	Mixed
IRF314	1.125	NSS
J4112	4.458	MIXED
JH59	1.75	NSS
JH96C	4.667	NSS
Ji53	3.208	NSS
Ji846	3.583	NSS
Jiao51	3.917	Mixed
K22	5.292	NSS
K22	9.333	NSS
L3180	3.208	MIXED
LG001	5.208	NSS
Liao138	4.167	NSS
Liao159	8.208	Mixed
Liao5114	3.167	Mixed
Liao5263	0.208	NSS
LK11	2.292	NSS
Lv28	1.75	NSS
LXN	7	NSS

LY042	7.417	NSS
M153	9.167	NSS
M165	9.208	NSS
M97	1.667	NSS
MO113	1.708	NSS
Mo17	2.75	NSS
Nan21-3	3.083	Mixed
P138	1.583	NSS
P178	3.375	NSS
Pool-1	7.583	Unknown
Q1261	4.583	NSS
Q319-BC6	4.167	Mixed
Qi205	2	Mixed
Qi319	7.333	NSS
R08	1.875	NSS
R15X1141	2	NSS
RY713	6.958	NSS
Ry729	3.25	Mixed
SC55	8.917	MIXED
Shen137	2	NSS
Si273	5.083	NSS
Si444	4.333	NSS
Sy1077	1.667	Mixed
Sy3073	4.5	NSS
Sy999	6.042	NSS
Tian77	2.435	Mixed
Tie7922	4.75	SS
TY11	2.333	MIXED
TY2	3.833	MIXED
TY3	5.458	MIXED
TY5	4.583	MIXED
TY6	8.083	MIXED
TY7	6.25	Mixed
TY-HZ4	4.167	Mixed
U8112	3.208	SS
W138	2.292	NSS
WH413	5.5	NSS
Wu109	1.792	Mixed
Xun971	7.75	Mixed
Y1382	9.348	Unknown
Y2348	4.167	Unknown
Ye478	6.833	Mixed
Ye515	6.667	Mixed
Ye52106	4.167	NSS
Ye8001	4.625	Mixed
Yu374	1.333	NSS
Yun46	8.542	TST
Z2018F	3.333	NSS
ZaC546	3.833	NSS
ZH68	5.167	SS
Zheng29	1.417	Mixed

Zheng30	1.708	NSS
Zheng35	3.792	Mixed
Zheng58	4.042	NSS
Zheng653	3.292	NSS
Zhi41	6.5	NSS
Zhong69	4.25	Mixed
Zi330	5.667	NSS
ZZ01	3.875	NSS
ZZ03	1.042	NSS

Table S2 Comparison of the protein IDs in V2 version and V4 version of maize B73

query_id_V2_version	subject_id_V4_version	% identity	alignment length	mismatches	gap opens	q. start	q. end	s. start	s. end	evalue	bit score
GRMZM2G400092_P01	Zm00001d019004_T001	62.46	341	28	7	112	360	106	438	7.00E-124	369
GRMZM2G431066_P01	Zm00001d012222_T001	100	150	0	0	1	150	1	150	1.00E-103	299
GRMZM2G319150_P01	Zm00001d035127_T001	94.96	139	7	0	176	314	84	222	2.00E-90	274
GRMZM2G042133_P01	Zm00001d047975_T001	71.6	669	49	7	18	601	12	624	0	922
GRMZM2G474575_P01	Zm00001d002960_T001	100	122	0	0	1	122	1	122	4.00E-81	244
GRMZM2G127591_P01	Zm00001d002941_T001	100	344	0	0	1	344	1	344	0	703
GRMZM2G002023_P01	Zm00001d012224_T014	100	641	0	0	1	641	1	641	0	1336
GRMZM2G428386_P01	Zm00001d002940_T003	100	1269	0	0	1	1269	97	1365	0	2644
GRMZM2G413887_P01	Zm00001d002959_T002	100	244	0	0	30	273	1	244	8.00E-180	501
GRMZM2G346263_P01	Zm00001d033654_T024	98.68	152	2	0	1	152	1	152	1.00E-102	314
GRMZM2G393070_P01	Zm00001d042633_T001	68.82	93	29	0	2	94	111	203	3.00E-39	134
GRMZM2G113569_P01	Zm00001d033578_T001	88.12	404	0	2	1	356	1	404	0	523
GRMZM2G393072_P01	Zm00001d012227_T001	100	119	0	0	1	119	1	119	4.00E-84	248
GRMZM2G096407_P01	Zm00001d012231_T003	100	484	0	0	1	484	1	484	0	972
GRMZM2G092817_P01	Zm00001d012228_T003	100	355	0	0	1	355	1	355	0	732
GRMZM2G309327_P01	Zm00001d047535_T001	99.46	186	1	0	1	186	1	186	3.00E-130	369
GRMZM2G122018_P01	Zm00001d033583_T001	100	225	0	0	1	225	1	225	1.00E-162	454
GRMZM2G108655_P01	Zm00001d047536_T001	100	1077	0	0	1	1077	1	1077	0	2189
GRMZM2G129065_P01	Zm00001d012223_T001	100	420	0	0	1	420	1	420	0	866
GRMZM2G114153_P01	Zm00001d002958_T001	100	216	0	0	1	216	1	216	1.00E-156	438
GRMZM2G092945_P01	Zm00001d012229_T001	100	461	0	0	1	461	1	461	0	937
GRMZM2G044557_P01	Zm00001d033655_T003	100	608	0	0	26	633	1	608	0	1231
GRMZM2G113990_P01	Zm00001d002954_T002	100	149	0	0	1	149	1	149	9.00E-102	294
GRMZM2G114149_P01	Zm00001d002955_T001	100	113	0	0	242	354	1	113	2.00E-77	239
GRMZM2G024131_P01	Zm00001d033580_T001	100	471	0	0	1	471	1	471	0	955
GRMZM2G171410_P01	Zm00001d002961_T002	90.26	380	0	1	1	380	1	343	0	699
GRMZM2G127581_P01	Zm00001d002942_T002	100	246	0	0	1	246	1	246	0	507
GRMZM2G085116_P01	Zm00001d033579_T001	100	190	0	0	1	190	1	190	6.00E-132	374
GRMZM2G092759_P01	Zm00001d012226_T001	99.63	273	1	0	1	273	1	273	0	556

Table S3 The expression levels of the candidate genes in 368 inbred lines

Inbred lines	GRMZM2G002023	GRMZM2G024131	GRMZM2G042133	GRMZM2G044557	GRMZM2G085116	GRMZM2G092759	GRMZM2G092817	GRMZM2G092945	GRMZM2G096407	GRMZM2G108655	GRMZM2G113569	GRMZM2G113990	GRMZM2G114149	GRMZM2G114153	GRMZM2G122018	GRMZM2G127581	GRMZM2G127591	GRMZM2G129065	GRMZM2G171410	GRMZM2G309327	GRMZM2G319150	GRMZM2G346263	GRMZM2G393070	GRMZM2G393072	GRMZM2G400092	GRMZM2G413887	GRMZM2G428386	GRMZM2G431066	GRMZM2G474575
150	1.88	0	3.7	2.5	0.03	0.56	2.98	6.81	1.93	4.44	3.62	1.41	0.08	2.62	0.09	6.21	4.71	3.54	1.93	0.79	0.02	0.2	0.32	0.19	0.01	0	0.23	0	0
177	2.35	0	5.07	2.46	0.2	0	3.34	3.66	2.6	4.26	3.12	0.17	0	1.2	0.2	1.81	4.46	2.55	1.77	0.47	0	0.08	0.09	0.3	0.23	0	0.2	0	0
238	3.22	0	3.55	1.58	0.67	0	3.18	3.46	0.13	4.65	3.01	0.06	0	1.3	0	7.07	4.62	2	0.42	0.18	0	0.04	0.15	0.25	0	0	0.15	0.05	0
268	2.56	0	1.25	1.78	0.67	0	3.7	3.35	0.48	0	3.67	0.1	0	2.24	0.08	5.08	4.71	3.13	0.84	1.74	0	0	0.47	0	0	2.7	0.08	0	0
647	2.74	0	5.19	1.53	0.26	0	4.43	3.71	0.12	0	3.16	1.19	0	0.4	0.31	2.05	4.09	2.64	0.19	0.67	0	0.12	0.37	0.43	0.58	0	0.03	0	0
1462	4.12	0	4.69	1.86	0.03	2.86	4.14	5.38	2.61	4.2	3.65	0.42	0	1.36	0	7.86	4.59	3.19	0.53	1.2	0	0.64	0.95	0	0.03	1.79	0.05	0	0
4019	2.29	0	4.51	0	0.07	0	6.3	8.19	4.91	0	2.69	0	0.16	2.44	0.82	5.31	3.98	2.98	0.16	3.19	0	0	0.59	0.21	0	0	0	0	0
5213	4.22	0	2.75	1.96	0.2	0	2.53	6.91	2.37	3.65	3.35	1.02	0	0	0	2.07	1.95	3.07	1.57	0.78	0	0.32	0.88	0	0	0	0.3	0	0
5237	4.87	0	4.79	2.28	0.07	0.59	4.28	7.62	0.66	3.94	3.79	1.63	0	1.68	0.05	1.07	4.6	3.87	2.53	1.63	0	0.14	0	0.34	0	0.84	0.28	0	0
7327	3.47	0	4.14	0.73	0.28	0	3.5	3.52	1.77	3.92	3.13	0.49	0	0.67	0	7.17	3.99	2.92	0.22	0.39	0	0.23	0.04	0.55	0	0	0	0.01	0
7381	3.97	0	5.86	2.13	0.27	0	3.07	3.95	0.28	3.81	3.62	0	0.03	0.94	0.12	2.86	2.62	3.52	1.16	0.85	0	0.17	0.29	0.28	0	0	0.04	0	0
8902	4.17	0	5.29	2.27	0.33	0	3.99	0.42	0.11	0.56	3.48	0.15	0	2.97	0	2.98	4.77	0.05	0.81	1.65	0	0	0.16	0	0	0	0	0	0
9642	3.35	0	1.4	2.27	0.13	1.2	4.35	4.81	0.13	4.52	3.5	1.62	0.07	1.61	0.81	7.18	4.63	3.56	3.11	0.56	0	0.23	0.84	0	0	3.27	0.01	0	0
81162	3.54	0	3.59	2.16	0.4	1.49	4.37	6.14	0.38	4.07	3.48	1.1	0	1.57	0	1.49	5.04	1.82	0.34	1.71	0	0	0.16	0	0	0	0.11	0.07	0
526018	4.04	0	4.43	2.53	0.96	1.79	5.66	9.37	0.99	4.08	3.76	3	0.23	2.82	0	0.34	7.96	5.02	3.96	3.51	0.02	0.04	0.64	0.5	0.05	1.6	0.27	0.62	0
05W002	2.66	0	0.89	2.47	0.08	3.06	3.51	7.09	1.55	4.65	3.49	1.21	0.05	2.14	0.22	5.15	2.18	1.19	2.79	1.57	0	0.33	1.32	0	0.03	0	0.06	0	0
05WN230	4.73	0	0.44	2.44	0.23	1.29	4.15	2.98	0.82	3.69	3.67	0.02	0	1.44	0	1.12	4.83	1.48	1.76	1.56	0	0.14	0.16	0.45	0.02	1.83	0	0.06	0
07KS4	3.85	0	6.31	2.4	0.05	0.14	4.22	4.55	1.2	3.65	3.3	0.16	0	2.39	0.22	0.54	4.73	3.31	1.37	0.86	0	0.04	0.52	0	0	1.48	0.09	0	0
18-599	2.94	0	4.97	2.21	0.09	0.37	5.55	5.59	2.81	2.94	3.54	1.72	0.03	2.99	0	4.95	4.18	2.61	1.88	1.87	0	0.03	0.68	0.62	0	0.82	0.07	0.07	0
303WX	3.14	0	0.42	1.82	0.05	0.66	4.89	6.07	1.31	1.95	3.89	0.97	0	2.13	0.23	1.3	6.8	3.19	2.23	0.59	0	0.05	1.06	0.46	0.02	1.47	0.08	0	0
4F1	4.54	0	4.7	2.62	0.26	0.16	4.38	7.34	0.07	3.49	4.07	1.12	0.03	2.03	0	5.32	4.77	3.73	0.74	0.73	0	0.43	0	0	0	0.98	0.15	0.35	0
7884-4HT	4.51	0	3.08	2.16	0.18	0	4.37	5.47	2.81	3.37	3.16	0.96	0.04	3.02	0.16	5.63	4.67	2.19	2.21	1.1	0	0.31	0.34	0.07	0	0	0.1	0	0
835A	2	0	5.75	2.38	0.64	0.37	3.05	5.9	0.53	4.21	3.69	1.35	0.02	0	0.26	6.18	3.27	3.58	2.11	0.59	0	0.19	0.23	0.21	0.19	0	0.18	0.38	0
835B	2.93	0	5.86	2.25	0.24	0.61	3.9	6.72	0.16	4.68	3.49	0.22	0	1.43	0.03	0.78	4.05	3.05	2.16	0.56	0	0.22	0	0.54	0.01	0.89	0.09	0.36	0
975-12	4.52	0	4.65	1.9	0.28	0	0.69	3.89	2.22	3.88	3.38	0	0.08	2.74	0.04	1.18	6.65	3.34	0.54	0.82	0	0.01	0.67	0.15	0	2.85	0.14	0	0
B11	3.08	0	6.07	2.61	0.26	0.01	3.79	7.2	0.2	0	3.73	1.8	0	2.09	0	1.45	4.14	3.41	2.16	0.57	0	0.17	0	0	0	0.85	0.07	0	0
B110	3.82	0	2.24	1.63	0.38	0.27	4.08	4.94	1.69	4.23	3.43	1.64	0	0	0.09	1.08	4.05	3.04	0	0.52	0	0.08	0.57	0	0	0	0.18	0	0
B111	4.17	0	3.01	2.37	0.02	0	3.09	2.36	0.08	4.28	2.82	1.51	0	2.25	0	5.63	4.96	3.33	0.66	0.86	0	0.14	0.09	0.14	0	0	0.07	0	0
B113	4.49	0	4.28	1.94	0.31	0.44	3.42	5.26	0.06	2.35	3.34	0.12	0.03	1.16	0	5.88	4.69	2.81	0.28	0.71	0	0.01	0.41	0.16	0	1.76	0.12	0	0
B114	4.99	0	5.17	2.02	0.18	2.1	3.29	4.78	0.69	4.47	3.2	1.04	0.07	2.92	0.3	7.53	4.63	2.83	1.18	0.78	0	0.37	0.83	0	0	0	0.05	0.08	0
B151	4.2	0	5.56	2.62	0.45	0	3.23	6.88	0.35	2.56	3.76	1.07	0.1	2.9	0.31	5.73	4.66	3.08	0.61	0.61	0	0.09	0	0.1	0	2.83	0.15	0.16	0
B73	2.78	0	0.36	1.7	0.04	3.18	3.9	5.56	0.37	1.66	3.15	1.04	0	0	0	2.6	2.37	3.23	1.03	0.91	0	0.26	0.38	0	0	0	0.09	0.04	0
B77	2.72	0	5.21	2.08	0.03	2.15	4.33	7.45	0.3	1.67	3.45	2.27	0	1.44	0.21	2.35	2.67	3.01	0.77	1.25	0	0.12	0.3	0.25	0	0	0.04	0	0
BS16	4.13	0	3.55	0.59	0.05	0	5.95	7.23	4.99	0	3.96	0	0	1.3	0	5.83	4.22	2.92	0.81	2.42	0	0	0.97	0.27	0	0	0	0.02	0
BY4839	4.36	0	5.72	0.58	4	0	4.02	3.7	0.12	4.04	3.33	0.12	0.04	1.31	0.05	1.29	4.81	2.4	0.48	0.57	0	0.25	0.07	0.23	0	1.02	0.04	0	0
BY4944	3.9	0	5.85	1.99	0.75	0	5.78	6.81	0.91	0	3.95	0.98	0	1.82	0	4.96	4.67	3.27	0.95	1.03	0.01	0	0.48	0.22	0	0	0.08	0.09	0
BY4960	4.55	0	5.43	1.6	0.1	0	3.1	5.19	2.35	0	3.93	0	0	2.66	0	5.01	4.85	2.4	0	1.37	0	0.02	0.12	0.19	0	0	0.02	0	0
BY804	3.63	0	5.64	2.24	0.38	0	4.78	6.41	0.69	3.18	4.23	1.95	0	1.45	0	5.14	6.51	2.89	0.34	0.99	0	0.01	0.8	0.52	0	0	0.06	0.02	0
BY807	4.4	0	5.98	0.7	0.04	0.22	0.47	4.92	0.03	3.74	4.67	1.92	0	0	0	6.81	4.81	3.34	1.5	0.42	0.01	0.02	0.64	0.25	0.18	0	0.02	0	0
BY809	2.58	0	5.55	2.53	0.01	0.76	2.1	7.66	2.72	4.36	4.12	1.68	0	1.45	0	5.81	3.21	2.92	2.52	1.21	0.01	0.22	0	0.02	0.02	0.13	0.08	0.02	0
BY813	2.79	0	5.52	2.21	0.2	0.4	3.22	5.07	1.88	3.92	3.66	1.43	0	0.63	0	8.04	4.8	2.92	1.77	0.75	0	0.17	0.65	0.45	0	1.21	0.04	0	0
BY815	2.07	0	5.48	1.9	0.04	1.83	3.92	5.9	1.04	4.17	3.74	1.45	0	0	0	5.31	4.21	2.57	1.22	0.33	0	0.1	0.29	0.24	0	0	0.03	0.01	0
BY855	2.77	0	2.12	2.05	0.65	0.01	3.9	4.2	2.91	3.16	4.05	1.97	0.05	2.01	0	4.79	4.97	2.76	1.93	0.78	0.01	0.02	0.26	0.57	0	0.02	0.12	0.03	0
BZN	3.65	0	6.2	2.36	0.01	0	2.5	4.38	0.08	2.85	4.17	1.24	0	0	0.05	5.26	6.2	2.74	0.35	0.51	0	0.19	0.04	0	0.03	0	0	0.01	0
CA47	4.36	0	5.77	2.08	0.05	0.07	3.01	5.09	0.17	3.29	3.57	1.6	0	1.21	0	0.81	8.06	3.51	1.09	0.66	0	0.23	0.21	0.21	0	0	0.05	0.01	0
CHANG3	1.83	0	5.03	1.01	0.22	0.68	3.02	1.09	0.14	2.6	3.21	0	0	1.37	0.47	4.82	4.05	2.94	0.08	0.05	0	0.23	0.56	0.04	0	0	0.1	0	0
CHENG698	3.82	0	5.16	2.62	0.32	0.29	4.08	4.85	1.82	4.49	2.81	1.55	0	0.85	0	5.46	4.6	3.69	1.01	1.63	0.01	0	0.54	0	0	0	0.08	0	0
CHUAN48-	4.35	0	6	2.96	0.14	0.53	2.82	2.71	1.41	3.95	3.61	0.14	0	0.38	0.84	3.4	3.46	2.08	0.67	0.79	0	0.14	0.35	0	0	0	0.03	0	0
CI7	3.19	0	5.39	1.23	0.43	0.5	3.86	5.65	1.67	3.96	3.22	0.19	0.03	2.42	0	7.13	4.38	3.43	0.33	0.42	0	0.01	0.61	0.59	0	0	0.05	0	0
CIMBL1	4.5	0	0.81	2.46	0.21	0	4.22	6.39	2.69	3.61	3.38	1.41	0	3.11	0	5.2	4.3	2.61	0.97	1.95	0	0.35	0.54	0.21	0	0	0.01	0.05	0
CIMBL10	5.44	0	3.5	2.24	0.03	0	3.6	5.61	0.12	0	3.85	0.84	0.02	0	0	0.8	4.11	3.08	1.39	0.47	0	0.12	0.96	0.38	0	0.25	0.23	0.17	0
CIMBL100	4.49	0	4.24	2.54	0.02	0.29	5.87	3.62	0.21	1.75	3.89	2.27	0.13	2.93	0.02	6.46	4.66	3.07	0.23	1.95	0	0	0.16	0.01	0	0	0.09	0	0
CIMBL101	3.34	0	4.16	2.33	0.08	0	3.16	7.52	0.12	3.25	3.86	0.01	0.14	1.9	0.03	6.4	6.04	3.57	2.03	0.96	0.01	0.03	0.22	0.37	0	0	0.05	0.04	0
CIMBL102	4.27	0	4.8	1.5	0.43	0.02	3.46	4.42	0.07	3.86	2.54	1.14	0	2.51	0	4.66	3.75	2.56	1.53	0.32	0	0.08	0	0	0	0	0	0	0
CIMBL105	3.22	0	4.77	2.2	0.14	0.67	4.42	5.6	0.2	2.96	3.79	1.75	0.16	1.73	0	6.33	4.22	3.63	0.24	0.3	0	0.15	0.38	0.2	0	0	0.08	0	0
CIMBL106	4.31	0	4.86	1.68	0.19	0	4.95	0.85	0	0	4.02	0	0	1.97	0	2.07	4.52	2.6	0.22	0.89	0	0	0	0.45	0	0	0	0	0
CIMBL108	4.16	0	4.09	2.05	0.82	0	4.84	3.81	0.1	0	3.87	0	0	0	0	0.73	4.07	2.6	0.51	0.39	0.02	0	0.6	0.12	0	0	0.09	0	0
CIMBL109	5.05	0	5.2	3.12	0.07	0	1.71	6.12	1.83	2.19	3.55	1.26	0	2.3	0	6.1	4.84	4.06	0.37	1.34	0.03	0.16	0.28	0.04	0	0	0.05	0	0
CIMBL11	5.54	0	0.79	2.74	0.06	0.01	3.73	5.32	0.88	2.14	3.97	1.43	0.02	2.5	0	5.51	4.7	2.8	1.36	0.9	0	0.22	0.33	0.22	0	2.51	0.1	0.05	0
CIMBL111	1.39	0	5.33	2.39	0.3	0.21	1.79	0.91	0.19	0	3.48	1.44	0.07	1.35	0.85	0.75	3.93	2.52	0.01	0.25	0	0.09	0.09	0.3	0	0	0.05	0	0
CML470	3.39	0	2.44	2.24	0.12	0.05	2.37	4.08	1.89	4.15	3.59	0.13	0.06	0.84	0	5.88	4.13	2.74	0.11	0.85	0	0.27	0.39	0.06	0	0.73	0.11	0	0
CIMBL113	3.05	0	4.61	2.37	0.27	0.11	4.57	2.74	0.41	2.58	3.43	1.61	0.11	2.52	0	1.47	4.03	3.02	1.96	0.88	0	0.02	0.08	0.54	0	0	0.11	0.18	0
CML479	4.61	0	4.85	1.24	0.17	2.97	3.92	7.72	0.1	0	4.01	1.18	0	1	0	1.47	4.52	2.3	0.49	0.71	0	0	0.42	0.73	0	0	0.01	0	0
CIMBL114	3.97	0	3.38	2.31	0.07	0.07	4.59	5.66	2.83	0	3.99	0.74	0	1.62	0.1	2.24	4.44	1.91	1.86	0.61	0	0.13	0.3	0.23	0	0	0.04	0	0
CML480	3.76	0	0.81	1.74	0.01	0.07	0.93	3.23	0.23	1.72	3.32	1.53	0	1.09	0	0.36	2.26	2.75	1.37	0.17	0	0.09	0.18	0.25	0	0	0.04	0.02	0
CML486	4.89	0	5.15	2.32	0.02	0	5.33	5.62	2.19	0.04	4.13	1.4	0.17	2.1	0.21	0.85	7.13	3.3	0.78	0.99	0	0	0.16	0.1	0	1.17	0.08	0.08	0
CML493	2.46	0	3.31	2.29	0.04	0	3.07	6.61	3.14	3.56	4	4.12	0.11	2.2	0	2.21	5.02	2.53	2.82	0.24	0	0.24	0.14	0.11	0	0.65	0.26	0	0
CML496	2.76	0	5.52	2.51	0.58	0	4.66	3.36	2.92	3.29	3.58	2.42	0.03	1.95	0	6.79	4.37	2.66	2.46	0.94	0	0.06	0.14	0.28	0	1.24	0.14	0	0
CIMBL115	1.36	0	4.95	1.87	0.04	0	5.3	4.03	0.25	0	3.77	0.22	0.41	1.47	0	1.69	4.27	3.52	0.34	0.61	0	0	0.36	0.64	0	0	0.02	0	0
CIMBL116	1.54	0	4.9	2.44	0.34	0.5	5.69	3.88	0.69	0	3.72	0.31	0	1.02	0.62	1.04	3.9	2.97	1.49	0.26	0	0.01	0.32	0.31	0	0	0.06	0	0
CML50	3.61	0	5.32	1.32	0.5	1.62	0	0	0.85	0	3.57	0	0	0	0	2.37	4.67	2.42	0.8	1.97	0	0	1.09	0.99	0	0	0	0	0
CIMBL119	3.1	0	0.25	2.16	0.05	0.51	3.64	5.76	3.52	0	4.04	1.57	0.03	1.46	0	1.37	2.3	3.16	2.39	0.66	0.02	0.04	0.64	0.17	0	2.79	0.04	0.04	0
CML69	3.98	0	5.14	2.25	0.13	0	2.85	2.89	1.56	3.5	3.82	0	0	0.99	0	1.22	4.85	2.54	0.36	0.34	0	0.24	0	0.07	0	1.53	0.08	0.05	0
CIMBL12	2.68	0	1.1	2.39	0.05	0.02	2.67	4.2	2.17	0	3.73	2.25	0.01	1.83	0	5.08	1.97	3.26	1.56	1.24	0	0	0.24	0.05	0	0	0.04	0	0
D863F	2.1	0	5.33	2.66	0.11	0.63	2.97	5.07	0.25	4.67	3.64	2	0.07	2.28	0.04	0.43	4.17	2.45	2.63	1.06	0	0.1	0.39	0.23	0.14	1.84	0.07	0	0

CIMBL120	0	0	5.22	0.15	0.13	0	0	2.43	0.6	0	3.79	2.11	0.06	1.6	0	2.68	4.67	1.84	0	0.79	0.01	1.09	0.44	0.05	0.07	1.15	0.16	0.01	0
DAN3130	4.67	0	5.32	2.24	0.15	1.33	4.26	3.76	2.86	4.15	3.38	0.02	0.06	2.83	0.07	1.75	5.46	3.37	0.48	1.25	0	0.13	0.02	0.11	0.01	0	0.07	0.02	0
CIMBL121	2.86	0	4.67	1.86	0.05	0.21	3	4.55	0.72	3.67	3.45	1.91	0	1.6	0.09	5.06	3.96	2.26	0.65	0.47	0	0.07	0.63	0.6	0	0	0.04	0	0
DAN340	4.24	0	4.66	2.51	0.16	0.46	4.04	7.51	2.09	4.05	3.68	1.94	0.13	3.56	0.08	0.82	6.67	2.95	1.33	1.15	0	0.5	0.86	0	0.01	2.52	0.03	0	0
CIMBL122	2.09	0	2.82	0.53	0.13	0.46	3.01	4.78	0.07	0	3.06	0.03	0.04	1.62	0	1.22	3.82	2.12	2.04	0.47	0	0.22	0.34	0.15	0	0	0.02	0	0
DAN360	4.41	0	1.13	2.26	0.03	0.5	3.81	6.51	0.27	2.1	3.23	1.04	0.05	2.35	0.05	1.46	4.83	3.41	0.72	0.45	0	0.17	0.72	0	0	0	0.11	0	0
CIMBL123	2.4	0	3.2	1.75	0.5	0.55	2.11	5.19	0.02	3.82	4.74	0.75	0	2.61	0.12	4.07	7.29	2.76	0.64	0.21	0	0.07	0.13	0.21	0	0	0.02	0.01	0
DAN4245	3.95	0	5.46	2.05	0.35	0.37	4.37	4.06	0.14	4.28	3.4	0.63	0.08	1.91	0.36	4.49	2.71	2.73	1.52	0.96	0	0.11	0.18	0.2	0	0	0.11	0	0
CIMBL124	2.66	0	6.18	2.43	0.02	2.51	3.83	3.87	2.62	0	4.24	2.42	0.18	3.33	0	4.92	4.93	2.99	0.75	1.67	0	0.01	0.23	0.96	0	3.5	0.04	0.03	0
DAN599	2.44	0	6	2.1	0.54	0	4.99	6.94	2.51	3.51	3.75	1.73	0.03	2.15	0.31	1.05	4.03	3.14	0.1	1.39	0	0.35	0.12	0.23	0.07	0	0.05	0	0
CIMBL125	2.35	0	3.99	1.96	0.07	1.51	3.1	7.05	1.07	3.55	3.61	1.05	0.06	1.26	0	4.97	6.72	2.44	0.28	0.36	0	0.21	0.37	0.57	0	0	0.02	0.06	0
CIMBL127	2.82	0	4.89	1.73	0.03	1.73	3.99	5.56	0.17	0	3.89	1.46	0	1.31	0	5.2	4.78	3.1	1.21	0.44	0	0.52	0.83	0.45	0.02	1	0	0	0
DH3732	4.56	0	6.07	1.95	0.08	0.9	2.23	6.36	1.49	3.72	3.55	0.51	0.02	3.14	0.02	6.68	7.59	3.54	0.72	0.8	0	0.08	0.1	0.1	0	0	0.12	0	0
CIMBL129	3.15	0	2.52	2.98	0.27	1.08	2.96	3.54	0	3.46	4.17	1.37	0.04	2.37	0	1.2	3.58	2.7	0.52	1.21	0	0	0	0.17	0	1.73	0.06	0	0
DONG237	3.45	0	0.63	2.22	0.35	0.88	3.2	4.64	1.65	4.45	3.4	1.49	0	0.46	0.6	7.56	3.05	2.7	0.44	0.46	0.03	0.07	0.44	0.66	0	0	0.05	0	0
CIMBL13	3.23	0	6.37	3.03	0.19	0	4.22	5.61	2.76	4.02	3.79	2.28	0.12	1.57	0.33	1.29	4.41	3.22	1.86	1.3	0	0.09	0	0.43	0	3.7	0.08	0	0
DONG46	0.03	0	4.32	1.96	0.51	0	1.79	7.18	2.23	2.93	3.22	0.46	0.02	0.14	0	2.18	6.39	3.46	0.5	0.5	0	0.01	0.21	0.07	0.07	1.38	0.19	0.06	0
CIMBL133	2.76	0	0.27	1.82	0.22	0.37	2.18	4.52	1.4	0	3.52	0.14	0.13	1.59	0	4.46	6.54	3.03	2.83	0.91	0.05	0.04	0.44	0.25	0	0	0.09	0	0
CIMBL137	4.23	0	1.26	0.27	0.64	0	4.36	4.12	0.73	2.91	3.56	0.16	0	2.99	0	1.87	4.25	3.9	0.56	1.68	0.03	0.01	0.33	0.2	0.66	0	0.13	0	0
EN25	2.26	0	4.24	2.61	0.34	0	4.29	3.12	0.53	1.95	3.96	0.96	0	2.21	0	6.79	6.71	1.44	0.47	1.23	0	0.1	0.34	0.66	0	0	0.06	0.01	0
CIMBL139	3.9	0	3.02	1.87	0.15	0.17	3.36	5.59	0.49	4.37	3.49	1.04	0	1.22	0	1.52	7.08	2.87	0.42	0.49	0	0.08	0.29	0.17	0	0	0.05	0	0
ES40	0.75	0	4.01	1.83	0.09	0.01	5.03	2.65	1.92	0	3.14	0	0	1.73	0.07	1.85	6.52	2.66	0.3	0.37	0	0	0.17	0.31	0.07	0.74	0.05	0.04	0
CIMBL140	3.74	0	3.82	2.2	0.53	0.41	2.3	3.07	0.12	0	3.36	1.16	0	0.75	0.11	5.51	3.17	2.75	1.92	0.27	0	0.19	0.67	0.14	0.8	0	0.02	0	0
FCD0602	2.62	0	0.7	2.14	0.12	0.13	3.05	5.27	0.09	3.49	3.22	0	0	1.9	0	4.33	5.49	2.98	1.1	1.2	0	0.01	0.14	0.5	0.25	0	0.01	0	0
CIMBL141	2.41	0	2.6	2.36	0.37	0.27	2.41	5.23	1.88	3.91	3.39	0.04	0	0	0	0.88	6.66	3.3	0.08	0.82	0	0.11	0.12	0.28	0	0	0.03	0.02	0
GEMS1	4.62	0	0.03	2.03	0.07	0	3.76	8.76	0.08	3.62	3.25	1.11	0	2.74	0	7.55	0.94	2.71	0.51	0.55	0	0.12	0.3	0.29	0	0	0.06	0	0
GEMS10	3.95	0	2.57	2.07	0.05	0.28	3.21	4.59	0.11	2.72	3.4	1.13	0	2.11	0	0.67	2.95	1.26	0.73	1.32	0	0.08	0.41	0	0.1	0	0.09	0	0
GEMS11	4.12	0	0.3	0.34	0.03	0	3.04	4.47	0.05	3.98	3.17	0.09	0	3.04	0	7.06	4.52	2.62	1.03	1.17	0	0.54	0	0	0	0	0.04	0.15	0
CIMBL142	4.59	0	2.69	2.23	0.52	1.72	3.16	6.95	0.04	4.35	3.58	0.17	0.03	0	0.04	0.88	4.44	3.62	1.03	0.45	0	0.04	0.35	0.18	0	0	0.24	0	0
GEMS13	4.8	0	3.75	0.72	0.15	0.07	3.31	5.16	1.55	0.01	3.38	1.45	0	2.51	0.17	4.74	4.64	3.29	0.52	0.34	0	0.2	0.1	0	0.06	0.5	0.13	0	0
CIMBL143	2.41	0	0.36	1.49	0.05	0.32	3.97	3.86	0.18	0	3.36	1.14	0	1.81	0.03	3.99	2.34	3.29	0.49	1.3	0.42	0.06	0.44	0.08	0	3.19	0.03	0	0
GEMS14	3.79	0	0.9	2.36	0.26	0	2.57	4.72	2.79	3.33	3.74	1.48	0.04	1.51	0	0.93	6.4	3.57	1.54	1.09	0	0.29	1.07	0.22	0.12	0	0	0.08	0
GEMS15	2.74	0	4.92	1.92	0.55	4.3	3.32	2.23	1.07	3.78	3.62	1.12	0	0.72	0.43	4.77	5.27	3.2	0.47	0.24	0	0.22	0.46	0	0	0	0.04	0	0
GEMS16	3.04	0	0.32	2.31	0.04	1.88	4	4.9	0.88	3.18	3.13	1.82	0	2.24	0.19	5.14	3.58	3.69	0.36	0.99	0	0.34	0.27	0	0.07	1.33	0.05	0	0
GEMS17	4.12	0	1.36	1.07	0.19	2.53	3.66	4.03	1.47	2.87	3.36	1.14	0	3.46	0	4.46	5.48	4.56	1.35	1.07	0	0.12	0.24	0.02	0	2.88	0.05	0	0
CIMBL144	3.27	0	2.17	0.84	0.62	0	4.25	4.2	0.11	0	3.25	0.75	0.03	0.38	0	4.42	5.67	3.94	0.52	0.65	0	0	1.19	0.09	0	0	0.09	0.03	0
GEMS18	3.73	0	3.95	0	0.22	0	2.79	4.2	0.59	3.72	2.55	1.35	0	2.85	0.1	4.06	4.15	2.18	0.64	0.87	0	0.17	0.21	0.31	0	0	0.11	0	0
GEMS19	2.1	0	0.64	0.94	0.05	0	3.92	6.07	1.67	0	3.68	0	0	3.33	0.05	5.1	5.6	3.02	0.6	1.4	0	0.01	1.17	0.76	0.17	0	0.13	0	0
CIMBL145	5.01	0	2.42	1.85	0.19	0.08	4.14	3.9	0.01	2.39	3.39	1.38	0.18	1.46	0.38	0.48	4.48	1.4	1.44	1.61	0	0.02	1.48	0.08	0	0	0.17	0.02	0
GEMS2	2.95	0	0.45	1.7	0.03	0.05	4.37	5.92	1.55	3.94	3.36	0.07	0.06	1.83	0.03	6.47	4.96	2.43	2.32	1.26	0	0.05	0.21	0.15	0	2.04	0.07	0	0
CIMBL147	3.63	0	3.97	1.13	0.01	0	4.48	4.54	0.02	0	3.5	1.96	0.23	1.48	0.07	3.3	4.47	2.89	0	1.56	0	0	1.68	1.4	0	0	0	0	0
GEMS20	1.86	0	1.06	1.95	0.11	1.15	4.14	5.56	1.64	0	3.45	1.69	0.07	0.56	0	1.51	1.77	2.81	0.4	0.67	0	0	0.45	0	0.63	0	0.12	0	0
CIMBL149	1.53	0	3.38	0	0.16	0	3.24	4.87	0.31	0	3.31	1.11	0	1.57	0	0.99	1.7	2.89	0.41	0.64	0	0	0.31	0.44	0	0	0.06	0	0
GEMS21	4.58	0	1.64	2.29	0.06	1.17	3.51	4.28	0.11	4.35	2.89	0.46	0.1	2.22	0.03	1.52	4.91	2.37	0.28	0.69	0	0.01	0.68	0	0	0	0.07	0	0
CIMBL15	3.83	0	0.26	2.3	0.36	0	2.33	5.96	1.03	3.96	4.02	0.03	0	0.81	0.29	0.85	2.62	3.11	1.07	0.72	0	0.08	0.15	0.27	0	2.56	0.06	0	0
GEMS23	3.63	0	0.51	0.98	0.05	2.74	5.23	5.9	1.43	0.09	3.45	1.44	0	2.09	0	4.87	4.91	3.05	0.39	1.29	0	0.23	0.54	0.12	0.03	1.46	0.01	0	0
CIMBL150	3.61	0	0.71	2.52	0.06	0.06	2.92	5.65	3.65	2.55	3.39	0.03	0	1.27	0	7.56	2.28	4	2.76	0.65	0	0.01	0.29	0.14	0	0	0.02	0.02	0
GEMS25	3.82	0	3.67	2.32	0.23	0.06	3.57	3.34	1.39	2.25	2.97	1.4	0.02	0	0	2.35	1.98	3.65	0.64	1.27	0	0.31	1.8	0.13	0	0.9	0.17	0	0
GEMS28	4.77	0	0.88	1.43	0.14	1.18	2.63	5.55	0.06	3.78	3.12	0.11	0	0.82	0	0.75	5.09	2.69	0.81	1.05	0	0.31	0	0	0.31	0	0	0	0
CIMBL151	4.31	0	1.14	1.34	0.04	0	4.66	8.19	1.5	0	3.89	0	0	2.32	0	0.8	5.05	3.73	0.2	1.39	0	0	0.76	0.42	0	0	0.04	0.01	0
GEMS29	0.79	0	1.04	2.36	0.69	1.34	3.78	4.34	1.11	4.01	3.04	0.4	0	0	0.02	0.68	2.36	4.4	1.48	0.8	0.01	0.22	0.08	0	0.08	0	0.08	0	0
CIMBL152	3.6	0	5.13	2.48	0.28	1.04	1.76	5.66	0.01	2.19	4.05	1.1	0	3.09	0.14	7.42	2.68	3.94	0.35	0.66	0	0.12	0.14	0.06	0	1.38	0.05	0	0
CIMBL153	4.05	0	0.77	2.53	2.94	1.41	5.24	3.81	0.28	0.01	3.39	0.53	0	1.07	0	0.56	4	3.46	1.52	0.85	0	0.01	0	0.14	0	0.41	0.13	0.02	0
CIMBL156	4.05	0	3.94	2.62	0.15	0	5.41	6.34	1.41	0.21	4.02	1.41	0.12	0.95	0	0.7	4.98	2.59	1.39	1.7	0	0.36	0.76	0	0	0	0.1	0	0
CIMBL157	3.87	0	0.86	2.46	0.04	0	2.72	7.87	0.59	0	4.21	1.69	0.11	3.73	0	0.44	6.69	3.22	2.17	1	0.08	0.19	0.96	0	0	1.99	0.37	0	0
CIMBL16	4.43	0	3.55	1.96	0.06	0	2.67	3.81	0.05	0	3.7	0.14	0.1	1.5	0	4.03	4.55	2.72	0.47	0.87	0	0	0.27	0.43	0	2.69	0.02	0	0
CIMBL17	4.79	0	5.18	0.85	0.21	0.43	2.29	5.23	0.75	0	3.06	0.68	0.02	0.26	0	1.41	7.25	2.44	0.76	0.51	0	0.25	0.38	0.2	0.06	0	0.12	0	0
CIMBL18	4.45	0	0.63	2.61	3.4	0	5.91	4.38	1.07	0	3.44	2.16	0.07	1.32	0	3.8	4.51	3.15	1.44	1.32	0	0.11	0.42	0.34	0	0	0.09	0.16	0
CIMBL19	3.62	0	4.29	2.05	0.05	0	3.83	8.29	0.26	0	4.01	1.42	0	2.29	0.07	0.37	2.69	3.01	0.38	1.03	0	0	0.24	0.14	0	2.22	0	0.02	0
CIMBL2	3.8	0	2.88	1.02	0.1	0	6.07	4.99	2.55	0	3.49	2.04	0.22	2.58	0	0.92	2.9	3.46	0.63	0.99	0	0	0.22	0.05	0	0	0.06	0	0
CIMBL21	4.95	0	3.24	2.6	0.37	0	2.77	3.63	1.56	0	3.33	1.22	0	0.63	0.02	3.51	7.14	3.29	1.51	1.01	0	0.22	0.28	0	0	0	0.06	0	0
CIMBL22	5.07	0	2.61	2.56	0.38	2.53	4.59	4.98	1.02	4.4	3.22	1.37	0.04	0.8	0.04	4.82	4.62	2.68	1.76	0.82	0	0.07	0.19	0.04	0	2.3	0.07	0	0
CIMBL23	4.96	0	5.49	2.6	0.87	2.66	5.89	5.3	0.06	3.94	4.24	2.25	0.11	1.74	0	0.2	4.32	3.4	1.87	1.36	0.01	0.14	0.71	0.14	0	2.32	0.02	0.13	0
CIMBL25	4.16	0	6.11	2.04	0.22	2.61	3.35	3.91	0.82	0	3.83	1.09	0.05	1.54	0	4.86	4.3	3.37	2.05	0.34	0	0.07	0.28	0.64	0	1.62	0	0	0
CIMBL27	4.16	0	3.37	2.06	0.07	1.01	5.67	4.77	0.19	3.3	3.83	1.93	0.17	0.93	0.16	0.33	4.21	3.85	1.04	0.67	0	0	0	0.09	0	0	0.2	0.56	0
CIMBL28	3.09	0	5.97	2.13	0.17	0.3	3.52	4.78	0.33	2.23	3.79	0	0	1.71	0	5.58	4.5	3.09	2.5	0.29	0	0.05	0.37	0.38	0	0	0	0	0
CIMBL29	4.65	0	5.07	2.35	0	0.38	4.43	4.86	1.15	0.11	3.87	0	0.06	1.97	0	0.78	4.88	2.33	0.45	0.8	0	0.07	0	0.02	0	1.3	0.28	0	0
GEMS3	4.26	0	0.51	1.62	0.04	0	4.41	4.26	1.72	4.34	3.6	0.05	0.04	0	0	0.85	3.52	2.99	0.58	0.78	0.01	0.1	0.04	0.16	0	0	0.03	0	0
GEMS30	2.99	0	0.85	2.23	0.04	2.79	4.3	3.83	2.28	3.32	3.07	1.4	0.05	1.25	0.04	3.37	7.38	3.62	0.6	0.95	0.02	0.14	0.57	0	0.07	1.13	0.01	0	0
CIMBL3	4.11	0	3.76	2.43	0.02	1.27	4.33	0.03	0.04	3.28	3.79	1.97	0.05	0.93	0.1	0.59	6.13	2.97	0.1	1.29	0	0.12	0.21	0.26	0	0	0.1	0.09	0
CIMBL32	4.6	0	3.68	2.54	0.06	0.24	3.9	5.32	0.66	4.37	3.63	0.91	0	0.7	0.2	0.4	4.46	4.18	0.97	0.38	0	0.37	0.4	0.31	0	0	0.2	0	0
GEMS31	3.53	0	5.38	3.06	0.23	1.76	4.75	4.3	1.26	0	3.56	1.13	0	1.81	0	3.59	2.39	3.7	0.47	0.94	0	0.13	0.17	0.09	0	1.34	0.16	0	0
GEMS32	4.39	0	4.41	2.68	0.14	3.12	5.4	2.62	1.93	4.17	3.53	1.89	0	1.17	0	0.35	7.51	2.49	0.43	2.03	0	0.85	0.53	0	0	0.7	0.08	0.17	0
GEMS33	4.21	0	3.18	1.96	0.51	0.66	6.64	4.93	3.18	2.17	3.75	1.66	0	1.68	0.25	4.85	4.51	3.48	1.63	0.84	0	0.04	1.4	0.87	0	0	0.03	0.14	0
GEMS35	2.62	0	4.88	1.8	0.17	0	3.01	5.06	2.25	4.6	3.44	0.53	0	1.06	0.4	5.96	4.73	2.45	1.61	1	0	0.21	0.19	0.48	0	0	0.04	0.12	0
CIMBL38	4.69	0	5.9	2.55	0.48	0.46	5.07	5.14	0.53	3.83	4.08	1.62	0.03	2.01	0.02	7.7	4.75	3.74	1.64	0.91	0	0.04	0.38	0.3	0	2.29	0.1	0.02	0
GEMS36	3.4	0	4.31	2.77	0.9	1.92	2.87	3.63	0.08	4.55	3.4	0.1	0	1.84	0	5.57	4.61	3.24	1.49	0.39	0	0.05	0.43	0.34	0	0	0.26	0	0
CIMBL4	2.38	0	2.79	1.7	0.11	0	3.85	1.1	2.23	3.26	3.09	1.29	0	0	0.31	0.63	3.55	2.62	0.83	0.29	0.01	0.01	0.26	0.13	0	0	0.05	0	0
GEMS37	5.15	0	3.36	2.84	0.69	1.15	5.04	7.69	3.94	2.64	3.89	2.45	0	2.64	0	8.41	5.27	2.19	1.37	1.19	0	0.14	1	0.29	0	2.59	0.04	0.32	0
GEMS39	3.29	0	0.31	2.44	0.34	0.67	3.91	3.41	2.67	4.21	3.32	1.33	0	1.64	0.14	7.3	3.41	3.3	2.74	1.18	0	0.14	0.66	0.27	0	0	0.09	0.01	0

GEMS4	4.95	0	0.68	2.69	0.35	0	4.93	3.83	0.92	4.29	3.39	2.63	0	3.07	0	6.24	4.45	3.53	0.5	0.99	0	0.24	0	0	0.11	0	0.07	0	0
GEMS40	1.64	0	1.87	1.13	0.03	2.15	4.58	6.65	2.95	0	3.66	1.09	0	1.44	0	5.01	4.64	3.12	1.28	0.88	0	0.48	1.3	0.57	0	0	0.03	0.06	0
CIMBL40	4.01	0	5.23	2.73	0.46	0.09	4.08	5.19	1.54	4.03	3.77	0.55	0.02	1.94	0.49	6.7	4.64	3.51	0.19	0.49	0	0.79	0	0	0	0	0.14	0	0
GEMS41	2.72	0	5.47	2.53	0.03	1.05	3.61	3.78	2.14	4.22	3.81	1.15	0	1.29	0.09	5.14	4.59	2.75	1.64	0.38	0	0.2	0.83	0.33	0	0	0.12	0.32	0
CIMBL42	3.06	0	2.81	2.52	2.87	0.4	0	2.86	0.07	4.3	2.9	0.14	0	0.57	0.03	0.35	6.7	3.17	2.07	0.53	0	0.27	0	0.28	0	0.25	0.03	0.05	0
GEMS42	4.91	0	3.62	1.95	0.44	0	4.33	5.07	0.72	4.14	3.61	0.2	0	0	0.12	5.44	5.08	3.22	1.81	0.96	0	0.04	0.54	0.3	0	0	0.13	0.02	0
GEMS44	4.97	0	0.17	2.01	0.26	0.46	1.95	6.07	1.85	4.35	3.59	0.76	0	0	0.27	3.94	4.07	3.41	0.52	0.78	0	0.22	0.39	0.22	0	0.66	0.04	0.09	0
CIMBL43	3.4	0	0.92	2.45	1.34	0	4.78	5.66	0.34	0	3.83	0	0	2.55	0	1.16	4.27	3.38	3.05	1.56	0	0.34	0.16	0.7	0	0	0.06	0	0
GEMS46	1.94	0	0.49	2.42	0.17	1.59	4.48	6.62	3.95	4.13	3.7	2.28	0.06	1.38	0.07	3.45	2.5	3.29	2.23	0.42	0	0.28	0.24	0.3	0	0.4	0.1	0.03	0
GEMS48	3.93	0	4.31	2.25	0.29	0	4	7.36	0.06	3.55	3.57	1.24	0	0.87	0	2.09	4.67	3.1	1.11	0.59	0	0.3	0.08	0.17	0	0	0.14	0	0
GEMS49	3.18	0	0.93	2.25	0.03	3.15	5.34	2.08	0.8	3.6	3.95	1.89	0.11	1.31	0.18	0.22	6.37	3.67	0.47	0.82	0	0.08	0.15	0	0	1.28	0.3	0.03	0
GEMS5	4.57	0	0.64	2.07	0.29	0	4.8	5.14	2.12	0	3.47	0.16	0.18	1.21	0	0.6	4	3.64	0.25	1.29	0	0.05	0.95	0.04	0	1.13	0.06	0	0
GEMS50	1.72	0	0.86	2.34	0.17	3.06	2.31	5.76	1.65	1.51	3.54	0.9	0	0	0.48	0.86	5.56	3.83	2.74	0.85	0	0.1	0.15	0	0	1.86	0.1	0.06	0
CIMBL46	3.01	0	5.05	2.49	0.24	2.26	4.46	6.75	3.67	3.9	3.57	1.36	0	0.7	0.2	4.18	7.97	3.93	0.9	1.1	0	0.02	0.15	0.37	0	0.71	0.14	0	0
GEMS51	3.1	0	0.77	0.99	3.65	0	3.85	4.85	1.9	4.47	3.32	0.08	0	0.62	0	0.66	3.02	3.19	0.04	0.68	0	0.01	0.68	0	0	0	0.45	0	0
CIMBL47	2.18	0	1.64	2.37	0.03	0.61	4.05	5.62	0.16	3.62	3.63	1.18	0	1.01	0	6.97	4.55	3.28	1.08	2.93	0.01	0.08	0.05	0.19	0.01	0	0.09	0	0
GEMS54	3.08	0	4.47	2.05	0.58	0.02	3.29	4	0.99	3.98	3.14	2.01	0.23	2.3	0	3.62	4.62	2.61	0.62	1.59	0	0.02	0.57	0.64	0	0	0.04	0	0
CIMBL48	4.81	0	5.1	2.76	0.02	0	1.82	3.89	0.47	3.65	3.64	2.05	0	1.74	0.04	0.87	6.71	3.71	3.61	1.35	0.02	0.09	0.27	0	0	1.51	0.28	0.02	0
GEMS55	2.92	0	0.66	2.15	0.3	0.89	2.93	3.77	0	3.03	3.19	0.04	0	0	0.44	6.28	4.92	2.02	0.04	0.8	0	0.01	0.44	0	0.06	0	0.01	0.04	0
CIMBL49	4.66	0	3.95	2.33	0.03	0.07	2.22	2.6	0.43	4.52	4.01	0.45	0.02	0.43	0	0.95	5.94	3.09	0.82	0.99	0	0.23	0.1	0	0	0	0.26	0	0
CIMBL5	3.43	0	3.54	2.1	0.07	0.06	4.21	1.76	0.2	0	3.43	0.17	0.04	0	1.21	0.38	4.45	2.75	0.61	0.54	0	0.13	0.45	0.08	0	0	0.01	0	0
CIMBL50	3.74	0	4.43	0.68	0.03	0.3	4.31	3.52	0.04	3.14	3.77	0.06	0	0	0	0.59	3.17	2.42	1	0.66	0	0.2	0.09	0.2	0	0	0.11	0	0
GEMS56	3.39	0	0.21	0.6	0.26	0	5.55	6.78	3.42	0	2.88	0	0.2	1	0.3	1.98	3.93	3.06	0.17	1.95	0.01	0	0.52	0.5	0	0	0.22	0	0
CIMBL51	4	0	5.87	2.64	0.04	0.35	4.59	4.6	1.94	3.88	3.34	0.82	0	1.53	0.6	4.74	2.88	3.3	0.65	1.18	0	0.12	0	0.02	0	3.31	0.12	0	0
GEMS58	3.19	0	5.11	2.29	1.78	0.03	2.21	4.05	0.64	3.77	3.44	1.72	0	0	0.93	5.44	4.64	2.43	3.14	0.82	0	0	0.26	0	0	0	0.2	0	0
CIMBL52	4.4	0	5.21	1.97	0.2	0.43	2.55	3.93	0.17	3.99	3.16	1.02	0	0	0.14	6.42	4.2	2.83	0.64	0.45	0	0.03	0.44	0.56	0	0	0.15	0.03	0
GEMS59	4	0	0.26	2.61	0.04	0	2.74	4.87	1.31	1.3	3.54	1.2	0.03	1.91	0	5.16	4.9	3.75	0.42	0.34	0	0.25	0.5	0.88	0	0	0.02	0	0
CIMBL53	2.06	0	3.24	0.8	0.06	0	5.38	6.75	0.3	0	4.1	0.03	0.19	0.58	0	7.68	4.68	3.53	0.67	0.38	0.01	0	2.23	0	0	0	0.25	0.45	0
GEMS6	3.29	0	5.18	1.65	0.09	2.28	3.46	6.03	1.02	0	3.41	0	0	2.47	0.27	7.5	4.91	3.65	0.48	0.86	0	0.08	1.03	0	0	0	0	0	0
CIMBL54	0.22	0	5.81	2.05	0.03	0.8	2.27	1.62	0	0	3.22	0.29	0	1.08	0.27	1.45	5.08	3.4	1.22	1.03	0.31	0.12	0.05	0.04	0.01	0	0.05	0.02	0
GEMS60	4.4	0	4.39	2.03	0.18	0	2.3	4.73	0.12	3.71	3.15	0.88	0	0	0	0.38	4.37	2.39	0	0.39	0	0.08	0.23	0.13	0	0	0.02	0	0
GEMS61	4.34	0	4.52	2	0.04	2.19	3.41	6.28	2.59	4.28	3.35	2.1	0	0.62	0	7.48	4.85	2.93	0.42	1.4	0	0.15	0.54	0.12	0.15	0	0.04	0.11	0
GEMS62	4.84	0	3.36	1.91	0.1	0	3.2	5.24	0.49	2.29	2.95	0.93	0	1.86	0	7.39	4.19	2.56	0.45	0.71	0	0.47	1.83	0	0	0	0.11	0	0
GEMS63	4.29	0	0.19	1.97	0.11	2.08	2.66	3.72	0.85	0	2.84	0.1	0.04	1.76	0	2.29	4.64	2.06	0.37	0.81	0	0.29	0.14	0	0.01	0	0	0	0
GEMS64	2.94	0	0.72	1.79	0.12	1.29	2.77	3.97	0.08	0.54	3.11	0.45	0	1.87	0.04	5.09	4.5	2.3	1.3	0.65	0	0.06	0.13	0.04	0.02	0	0.04	0	0
GEMS65	4.68	0	0.24	1.07	0.2	0.69	2.58	6.37	0.15	4.52	3	0.61	0	0.71	0.45	2.11	3.46	2.07	0.5	0.64	0	0.02	0.8	0	0.05	0	0	0	0
LIA05114	2.73	0	3.84	2.18	0.85	0.24	4.03	4.82	2.19	4.43	3.65	1.2	0.06	3.07	0.38	0.69	5.96	3.34	1.91	1.32	0	0.11	0.43	0	0	0.96	0.03	0	0
GEMS66	4.58	0	3.41	2.3	0.06	0.66	2.48	6.23	0.97	4.43	3.49	0.88	0.11	0.95	0.21	5.21	4.95	3.3	2.34	1.73	0	0.17	0.7	0.21	0.04	0	0.21	0	0
GEMS9	2.51	0	5.6	1.99	0.05	3.4	2.24	5.38	2.59	4.05	3.03	0.77	0	2.79	0	4.4	3.19	3.11	1.18	0.7	0	0.19	0.61	0	0.49	0	0.14	0.01	0
LIA05262	2.09	0	5.24	2.57	0.53	0.15	5.39	4.32	0.06	3.89	4	1.63	0	3.67	0.09	0.47	4.74	2.26	2.3	1.82	0	0	0.27	1.29	0	0	0.22	0.04	0
GY1007	2.55	0	4.82	2.51	0.13	0.32	3.83	4	0.08	2.62	3.66	0.88	0.06	2.68	0.61	4.42	7.29	3.19	3.06	0.73	0	0.23	0.18	0.19	0	0	0.11	0	0
GY1032	3.45	0	3.95	2.45	0.34	0.36	3.32	4.72	6.15	3.97	3.25	1.6	0.02	3.63	0.26	0.42	6.72	4.13	0.86	1.85	0	0.19	0.05	0.05	0	0	0.11	0	0
LIA05263	4.37	0	5.53	2.31	0.52	0	5.01	2.03	0.27	0	3.78	0	0	2.13	0.38	1.18	2.94	0	0.66	1.65	0	0	1.6	0.63	0	0	0.18	0	0
CIMBL55	4.31	0	4.52	2.21	0.08	0.2	5.34	5.21	3.29	2.5	3.93	1.34	0.15	1.84	0	7.73	4.51	2.78	1.73	0.99	0.05	0.14	0.31	0.17	0	1.66	0.06	0.55	0
GY386	3.78	0	1.29	2.08	0.28	0.1	4.68	3.19	0.03	1.1	3.13	1.09	0	0.75	0	5.29	7.1	2.58	0.26	0.53	0	0.14	0	0.13	0	0	0.03	0	0
LK11	3.61	0	3.03	1.88	0.26	0.17	3.9	2.18	0.22	3.65	3.06	0.77	0	1.75	0	6.78	3.93	2.73	0.42	0.65	0	0.16	0	0.05	0.02	0	0	0.38	0
CIMBL56	3.67	0	5.07	0.63	0.04	0.68	2.51	4.73	1.33	3.83	3.67	0.03	0	1.15	0.04	5.06	4.73	3.11	1.93	0.59	0	0.24	0.36	0.16	0	1.51	0	0.01	0
GY462	3.9	0	1.14	2.68	0.19	2.3	5.57	4.53	0.25	1.28	3.55	1.2	0	1.1	0	4.49	4.81	3.26	1.49	1.23	0	0	0	0	0	0	0.07	0	0
CIMBL58	4.31	0	0.49	2.3	0.42	0.94	4.56	3.82	0.36	0	3.17	1.49	0	1.36	0	1.96	3.18	2.79	0.3	0.96	0.02	0.46	0.22	0.1	0	0	0.04	0.02	0
GY798	4.47	0	0.09	2.03	0.01	0	4.05	4.47	0.56	4.27	4.12	0.71	0	0	0	4.92	4.6	2.76	0.27	0.25	0	0.14	0.42	0.75	0	0	0.02	0	0
LV28	4.34	0	4.09	2.15	0.23	0	4.53	9.03	1.43	0	3.4	0.91	0	0.72	0.99	4.96	5.81	2.96	0.45	1.69	0	0.02	0.65	0.2	0	0	0.02	0.08	0
CIMBL59	2.72	0	4.64	2.23	0.11	0.16	4.35	4.67	0.07	4.05	3.14	0.05	0	0.87	0.46	2.13	4.57	2.82	0.69	1.1	0	0.11	0.21	0.08	0	0	0.09	0	0
GY923	3.87	0	5.62	2.22	0.06	2.7	5.21	4.87	0.77	3.58	4.3	1.31	0.03	3.34	0.58	0.41	3.12	3.3	1.13	0.82	0	0.06	0.4	0.32	0.43	0	0.08	0	0
CIMBL6	4.18	0	3.98	1.71	0.05	2.53	6.11	1.92	0.62	4.08	3.8	1.36	0	1.76	0.63	3.04	4.83	3.33	2.23	1.44	0	0.31	0.53	0.39	0.05	0	0.05	0	0
LX9801	3.49	0	5.17	2.16	0.07	0.44	2.19	2.41	1.61	3.49	3.71	0.96	0	0	0	1.99	4.63	2.95	1.65	0.44	0	0.03	0.13	0.03	0	0	0.12	0.01	0
HTH-17	2.83	0	5.13	2.5	0.24	0.16	3.62	2.5	1.71	3.19	3.61	1.42	0.09	3.69	0	4.8	4.48	3.18	0.28	0.49	0	0.43	0.19	0.1	0.02	0	0.27	0.06	0
CIMBL60	3.79	0	4.26	1.74	0.09	0.15	2.42	4.23	0.07	3.81	2.86	0.84	0	0.52	0.04	4.57	4.24	2.17	1.63	0.08	0.08	0.19	0.29	0.07	0	0	0.04	0	0
LXN	1.99	0	4.12	1.9	0.05	0	4.75	2.2	0.01	0	3.68	0.05	0	0.67	0	4.59	4.47	2.12	0.14	1.84	0	0.24	0.75	0.28	0	0.87	0.16	0	0
HUA83-2	4.48	0	0.28	2.05	0.34	0.16	5.6	0.9	0.67	3.97	3.35	0	0.03	3.27	0.61	8.13	4.67	2.8	0.43	1.8	0	0.18	0.25	0.68	0	3.36	0.04	0.14	0
CIMBL62	2.94	0	0.57	0.45	0.19	0	5.92	7.69	3.2	0	4.23	0.67	0	0.53	0	6.75	4.62	3.92	0.32	2.14	0	0	0.18	0.47	0	0	0.02	0.08	0
HYS	4.91	0	3.92	1.98	0.04	0.43	2.46	3.74	0	4.33	3.52	0.58	0.07	1.48	0	4.51	4.43	3.04	1.91	0.53	0	0.2	0	0	0	0	0.34	0	0
LY042	2.46	0	5.22	2.43	0.05	0.15	4.31	3.2	0.71	4.7	3.29	1.43	0	1.8	0.01	1.22	6.36	3.1	1.78	0.68	0	0.17	0.07	0.13	0	0	0.09	0.01	0
CIMBL63	4.34	0	1.1	0	0.11	0	4.94	0	0.88	3.73	3.64	0.18	0.02	1.16	0.22	1.37	4.45	2.75	1.7	1.18	0	0.08	0.31	0.17	0	0	0.18	0	0
CIMBL66	4.39	0	3.42	2.16	0.18	0.91	2.58	5.08	2.25	4.47	4.06	1.35	0	0.93	0	5.08	5.12	2.73	1.78	0.44	0	0.39	0.18	0	0	2.17	0.04	0	0
HZS	5.15	0	3.58	2.05	0.3	0.3	2.15	4.73	1.79	4.3	3.79	1.06	0	2	0	1.97	7.89	2.72	0.98	1.37	0	0.56	0	0.01	0.03	0	0.14	0	0
M153	3.58	0	3.82	2.15	0.08	0.27	3.92	2.03	1.46	1.99	3.37	0.08	0.03	0.53	0	4.01	2.81	2.6	0.44	0.27	0	0.01	0.08	0.53	0	0	0.01	0	0
CIMBL68	5.47	0	3.02	2.09	0.38	0.22	3.6	6.78	0.48	4.08	4.16	1.64	0.13	1.18	0	5.15	4.68	2.94	1.93	0.51	0.01	0.15	0	0	0	0	0.03	0	0
IRF314	5.13	0	3.87	2.2	0.35	0.01	4.48	6.01	2.61	0	3.6	0.01	0	1.75	0.1	5.98	3.42	3.21	0.76	1.69	0.01	0	0.49	0.8	0	0	0.14	0.03	0
CIMBL69	3.36	0	3.38	2.39	0.16	0.64	3.33	4.67	2.1	4.29	3.76	1.72	0	1.38	0	5.48	3.19	2.88	2.95	0.56	0	0.02	0	0	0	1.4	0.04	0	0
M97	4.35	0	3.45	1.79	0.41	0	3.38	3.88	0.13	3.56	2.82	0	0	0	0	5.45	2.43	1.95	1.03	0.43	0	0	0.16	0.2	0.12	0	0.04	0	0
J4112	2.81	0	0.98	1.88	0.06	0.97	3.15	5.23	1.59	4.44	3.47	1.11	0	1.89	0	2.31	4.2	3.38	0.81	0.8	0	0.96	0.55	0	0	0.82	0.01	0	0
CIMBL7	4.87	0	4.12	1.83	1.2	0.28	3.12	4.46	1.02	0	3.42	0	0.16	2.7	0.67	1.2	3.11	3.48	0.3	1.19	0	0.26	0.14	0.29	0	2.13	0.07	0	0
JH59	4.08	0	4.97	0.36	0.04	0.01	4.17	5.89	0.04	0	3.69	0.01	0	0.97	0.04	7.93	4.94	3.21	0.73	0.93	0	0	0.29	0.64	0	0	0.06	0	0
CIMBL70	3.03	0	5.54	1.01	0.05	0	4.48	5.7	0.33	0	3.98	0.05	0.14	0	0	0.65	4.85	0.18	1.79	1.14	0.01	0	0	0	0	0	0	0.09	0
MO113	4.78	0	5.04	2.05	0.05	0.02	4.67	4.03	0.39	3.42	3.4	0.8	0	2.52	0.05	5	4.5	2.51	0.89	1.28	0	0.15	0	0	0	0	0.05	0.08	0
JH96C	4.1	0	5.47	2.06	0.16	0.7	3.74	4.37	0.43	3.81	3.44	1.1	0.06	2.55	0.06	1.72	4.85	3.7	0.72	0.65	0	0.02	0.38	0.1	0	2.52	0.04	0.1	0
CIMBL71	3.08	0	3.25	2.33	0.43	2.34	3.25	4.89	0.39	0	3.98	0	0.08	1.12	1.12	6.89	4.47	3.45	0.21	0.56	0	0.23	0.4	0	0	0	0.12	0.19	0
MO17	3.2	0	5.93	2.35	0.06	1.73	1.88	4.72	0.08	1.37	3.48	1.19	0	1.2	0.44	5.36	5.24	3.03	0.41	0.44	0	0.15	0.1	0.34	0	1.75	0.05	0.01	0
JI63	3.34	0	4	1.87	0.28	0.02	2.99	2.92	0	4.47	3.02	0	0	1.05	0	1.18	6.37	2.69	0.77	0.19	0	0.18	0	0.04	0	0	0.09	0.03	0
CIMBL74	3.78	0	5.75	3.2	0.74	0	4.75	4.77	0.43	4.17	4.21	1.9	0.24	1.85	0.02	1.49	7.45	3.81	1.32	1.36	0	0.14	0.64	0.4	0	0	0.13	0.26	0

CIMBL75	4.48	0	5.6	2.61	0.09	0.4	4.01	4.46	0.15	3.05	4.01	0.02	0.23	1.37	0	1.43	7.43	2.43	1.91	0.37	0	0.01	0.07	0.49	0	0	0.07	0	0
JI842	3.84	0	5.37	1.57	0.13	0	3.9	3.84	0.18	4.36	3.31	0.92	0	1.33	0.38	6.3	4.72	3.16	0.76	0.61	0	0.11	0.05	0.38	0	1.44	0.21	0.09	0
NAN21-3	2.99	0	3.76	1.24	0.09	2.63	3.02	4.07	1.61	4.13	2.92	0.07	0	0.47	0.18	5.15	4.17	2.49	0.59	0.45	0	0	0.92	0	0	0	0.17	0	0
CIMBL77	3.29	0	3.46	2.8	0.04	0.59	5.56	6.07	0.87	0.19	3.63	1.46	0.1	1.67	0.05	5.51	7.3	3.47	2.65	0.68	0	0.04	0.12	0.1	0	0	0.06	0	0
JI846	4.6	0	3.31	1.87	0.21	0	3.74	2.14	0.62	3.35	3.52	0.99	0	2.59	0	4.52	3.64	2.05	0.51	0.47	0	0.27	0.49	0.14	0	1.6	0.02	0	0
P178	0.02	0	4.42	1.16	0.01	0	1.86	0	0.02	0	3.83	0	0	0	0.05	0.77	4.15	0.88	0.22	0.9	0	0	0.14	0.65	0.14	0	0.07	0.15	0
CIMBL79	2.97	0	2.83	2.13	0.03	0	5.05	2.23	0.05	3.65	3.47	2.5	0.18	1.83	0.31	6.45	4.83	2.49	1.69	1.14	0	0.08	0.07	0.27	0	0	0.09	0.01	0
JI853	5.24	0	3.75	2.25	0.05	0.02	1.29	2.4	1.27	4.31	3.53	1.42	0	1.8	0	2.51	2.48	2.84	0.83	1.25	0	0.23	0.53	0	0.43	0	0.2	0	0
Q1261	3.96	0	5.55	2.05	0.06	0	4.77	2.94	0.49	2.84	3.39	1.17	0.26	2.38	0.12	0.9	6.75	2.64	2.15	0.5	0	0.05	0.08	0.68	0	0	0.21	0	0
CIMBL81	3.85	0	2.35	2.24	0.56	0.11	3.71	4.62	1.7	2.54	3.54	1.57	0.13	1.93	0	4.62	6.41	2.62	2.55	0.93	0	0.08	0	0.16	0	2.3	0.07	0	0
CIMBL82	3.53	0	3.42	0.88	0.04	0.27	3.46	4.74	3.15	2.3	3.61	1.74	0.03	0.55	0	1.4	2.4	2.84	1.66	0.35	0.01	0.22	0.29	0.05	0	1.76	0.06	0	0
QI205	3.53	0	5.52	2.64	0.01	0	5.25	2.87	1.68	4.61	4.31	1.57	0	1.31	0.54	5.51	4.87	3.89	0.64	1.28	0	0.31	0.27	0.34	0.02	0	0.11	0	0
CIMBL83	4.07	0	4.58	1.78	0.08	0.09	3.91	4.28	0.36	3.37	3.32	0.29	0	0	0	5.07	4.1	2.7	0.12	1.13	0	0.24	0	0.13	0	0	0.04	0	0
R15	4.35	0	4.84	0.88	0.01	0	4.54	5.6	0.08	3.15	3.5	0	0.03	2.08	0	0.8	7.11	2.86	0.14	0.9	0	0.15	0.27	0	0	0	0.15	0	0
CIMBL84	0	0	4.97	2.33	0.09	0.4	0	2.36	0	0	4.63	0	0	0	0	4.81	0	0	0	0.89	0	0	0	0	0	0	0.05	0	0
R15X1141	5.28	0	4.84	2.86	0.08	0	3.61	6.81	0.1	3.99	3.44	1.32	0	2.2	0	5.74	5.01	2.9	1.42	0.16	0	0.04	0.2	0	0	0	0.11	0	0
JIA051	1.92	0	1.73	2.15	0.05	0	4.57	6.6	0.94	2.49	3.92	2.55	0	2.29	0	0.38	6.5	3.31	1.9	0.78	0	0.01	0.36	0.25	0	1.83	0.03	0.2	0
JY01	3.61	0	3.48	1.4	0.03	0	6.17	6.34	0.12	1.76	3.81	1.09	0	2.66	0.02	4.07	4.75	3.35	0.5	1.53	0	0.05	0.37	0.29	0	0	0.06	0.05	0
RY713	4.57	0	4.5	2.02	0.13	0.35	5.36	5.02	0.19	0	3.71	0.89	0	0.8	0.5	5.5	4.12	3.24	0.71	1.37	0	0.01	0.45	0.58	0	1.81	0.08	0	0
CIMBL86	4.77	0	5.17	2.29	3.07	0.01	3.05	3.54	0.05	0	3.05	1.98	0	1.27	0.04	4.79	2.15	3.22	0.68	0.38	0	0.03	0.12	0.01	0	0	0.15	0	0
K10	3.68	0	5.66	1.83	0.7	0.11	4.24	4.53	0.41	1.52	3.07	0	0	1.97	1.66	7.63	5.16	2.83	0.14	0.48	0	0.04	0.77	0.66	0	0	0.03	0.32	0
CIMBL87	4.61	0	3.25	2.04	0.75	0	0.22	3.68	0.79	3.16	4.02	0.9	0	1.34	0	4.99	4.11	3.7	0.62	0.75	0	0.2	0.27	0.08	0	0	0	0	0
RY729	2.86	0	4.74	2.19	0.2	1.01	5	2.67	2.49	3.39	3.78	0.95	0	0.21	0	5.34	4.69	2.98	0.79	1.45	0	0.11	0.78	0.2	0	0	0.1	0.1	0
CIMBL88	2.41	0	3.88	2.77	0.04	0.49	4	5.35	1.71	3.59	3.48	1.03	0	2.15	0	2.19	5.59	3.16	0	2.05	0	0.01	0.84	0.11	0	0	0.1	0	0
K12	4.5	0	4.19	2.09	0.17	0.84	4.58	6.66	1.88	1.72	3.68	0.86	0	3.03	0	2.9	5.08	3.13	0.09	0.77	0	0.06	0.55	0.75	0.01	0.88	0.02	0	0
CIMBL89	4.08	0	0.42	1.75	0.04	0.06	1.6	3.36	0.01	4	3.7	0.63	0	0.59	0.2	4.52	3.82	2.07	0.45	0.55	0	0.05	0	0.12	0	0	0.02	0	0
K14	4.82	0	4.11	2.64	0.05	0	5.87	4.41	2.13	3.48	4.06	1.02	0.14	1.45	0	1.3	4.58	2.98	1.69	0.43	0	0.14	0.76	1.29	0	0	0.06	0.01	0
S22	3.28	0	3.58	1.84	0.05	0.16	3.79	6.02	1.23	3.15	3.77	1.18	0	1.57	0.03	0.32	2.84	3.76	0.96	0.54	0	0.02	0.42	0	0	0	0.14	0	0
CIMBL9	4.98	0	3.14	1.91	0.29	0	2.78	4.41	0.62	2.11	3.43	1.22	0	0.9	0	6.27	4.17	2.81	1.69	0.79	0	0.03	0.51	0.3	0	0	0.1	0	0
K22	4.8	0	3.53	1.97	0.04	0	5.99	5.92	0.49	0	4.29	0	0.15	0.88	0	1.1	5.25	2.88	0.07	1.38	0	0.47	0.12	0.13	0	1.02	0.01	0	0
CIMBL90	4.2	0	3.28	1.78	0.15	0	3.32	4.43	0.03	0	3.86	1.88	0	1.59	0	3.61	4.6	2.7	1.81	1.03	0	0	0.94	0.17	0	0	0.04	0	0
CIMBL91	3.79	0	0.72	2.23	0.01	1.67	4.46	4.55	0.43	4.55	3.93	1.6	0	2.22	0	3.81	4.84	3.92	3.59	0.8	0	0.15	0	0.63	0.03	2.85	0.05	0.02	0
L3180	3.2	0	5.01	1.84	0.05	2.55	4.06	5.85	3.48	2.05	3.18	0.96	0.03	1.6	0.15	7.28	3.08	3.28	0.2	0.46	0	0.06	1.38	0.07	0.03	0	0.01	0.02	0
S37	2.92	0	4.88	2	0.13	0.43	3	5.02	0.08	2.37	3.73	0	0	1	0	0.49	7.25	2.77	0.61	0.95	0	0	0.33	0.18	0	0	0.06	0.05	0
CIMBL92	2.7	0	0.2	1.09	0	0.68	3.45	1.21	1.09	3.56	3.64	0.27	0	1.58	0.04	2.91	4.64	0	0.37	0.82	0	0.86	0.99	0.18	0	2.4	0.02	0	0
LG001	3.3	0	5.27	2	0.56	0	3.9	2.78	1.34	2.8	3.55	1.46	0.13	2.69	0.36	1.58	7.19	3.85	1.23	0.73	0	0.16	0.06	0.15	0	1.56	0.07	0	0
CIMBL93	3.91	0	3.72	1.83	0.02	0.01	4.29	6.25	1.65	1.95	3.67	0.04	0	1.17	0.02	1.73	6.37	3.07	0.32	1.55	0	0.15	0.18	0.31	0	0	0.09	0	0
SC55	3.06	0	1.25	2.28	0.14	0	4.45	6.42	0.31	0.04	3.38	0.18	0	0	0.03	4.94	4.49	3.61	0.39	0.81	0	0.05	0.48	1.14	0	0	0.03	0.03	0
LIA0138	4.54	0	4.31	2.32	0.29	0.58	3.56	4.91	2.21	4.4	3.15	0.84	0	2.71	0	6.41	4.9	3.86	1.93	1.32	0	0.13	0.63	0	0	0	0.18	0.01	0
CIMBL94	4.98	0	0.21	1.58	0.11	3.16	4.13	2.39	0.77	0	3.86	0	0	0	0.42	0.82	5.68	3.11	0.31	0.36	0	0.02	0.07	0	0	0	0.17	0	0
SHEN5003	4.42	0	3.15	2.18	0.2	0	3.95	6.16	0.01	2.19	3.39	2	0	0	0	3.23	4.69	2.05	1.4	0.6	0	0.02	0.5	0.57	0	0	0.03	0.02	0
LIA0159	4.37	0	5.38	2.26	0.25	0.42	3.77	4.21	1.4	4.23	2.98	1.08	0	1.13	0.02	4.88	3.7	3.5	0.82	0.46	0.01	0.46	1.13	0	0.04	0	0.12	0.04	0
CIMBL95	3.91	0	3.42	1.66	0.08	0.41	3.29	5.63	0.42	4.24	2.98	0.02	0	2.3	0	6.91	4.76	2.71	0.54	0.91	0	0.12	0.14	0.14	0	0	0.03	0	0
SI273	4.71	0	4.2	2.64	0.01	0	3.92	3.54	1.92	4.62	3.64	0.27	0.11	0	0.46	4.65	5.21	3.31	2.24	1.23	0	1.99	0.42	0.21	0.42	0	0.01	0.02	0
CIMBL96	4.37	0	3.18	2.13	0.08	0	4.13	0.12	1.69	3.09	3.34	0.17	0	0.91	0.03	1.38	4.18	2.86	0.87	0.79	0	0.34	0.28	0.21	0	0	0.05	0	0
CIMBL98	3.61	0	2.96	0.47	0.01	0.01	1.76	0.44	2.41	3.3	3.27	1.97	0	0.65	0.39	1.13	2.41	2.51	0.1	0.43	0	0.05	0	0.38	0	0	0.01	0	0
SI434	3.16	0	2.87	0.73	0.18	0	3.82	3.43	0.08	0	3.57	0.09	0	2.18	0.15	1.02	4.49	3.12	1.34	0.97	0	0	0.02	0.4	0.06	0	0.11	0.04	0
CML324	3.72	0	6.5	2.97	0.14	2.55	3.99	5.9	0.57	4.33	3.7	2.35	0	0	0.07	5.04	4.52	2.73	1.79	0.87	0.04	0.08	0.28	0.32	0.78	0	0.13	0.05	0
CIMBL99	3.3	0	4.75	2.14	0.1	0	1.34	6.23	0.08	4.19	3.52	1.79	0.15	2.7	0	0.87	2.77	3	1.2	0.79	0	0.17	0	0	0	2.24	0.19	0.13	0
SI446	4.25	0	2.94	1.58	0.24	0	4.16	6.7	0.29	3.46	3.02	0.62	0	1.44	0	7.64	4.71	2.22	0.72	0.54	0	0.46	0.49	0.16	0	0	0.03	0.53	0
CML325	4.73	0	0.41	2.35	0.07	0.36	3.36	4.99	0.66	0	3.91	3.46	0	2.7	0	0.16	6.49	2.47	0.2	1.42	0	0.22	0.22	0.25	0.17	1.84	0.11	0	0
CML114	4.24	0	0.23	0	0.4	0	5.92	8.5	5	0	3.86	0	0	3.73	0	6.36	4.47	3.03	0	2.46	0	0.02	0.14	0.06	0	0	0.09	0	0
CML327	4.69	0	0.24	2.86	0.33	0.67	2.28	4.83	2.39	0	3.79	0	0.07	0	0	0.14	4.71	2.26	2.22	0.49	0	0.3	0.06	0.74	0	0.69	0.1	0.02	0
CML115	4.47	0	0.57	2.57	0.05	0.76	5.06	7.46	3.29	0	4.56	0.6	0	1.39	0	6.64	4.96	3.91	3.15	0.39	0	0.15	0.34	0.14	0	0	0.03	0	0
SW92E114	4.01	0	6.06	2.45	0.12	1.74	4.42	5.82	3.7	2.7	4.44	1.87	0	2.28	0.07	0.25	6.9	2.97	2.07	1.05	0	0.2	0.34	0.44	0	2.12	0.08	0	0
CML116	5.06	0	0.22	2.19	0.34	0	4.36	2.6	1.51	3.98	3.27	0.04	0.08	0.37	0	1	6.57	3.57	0.35	0.71	0	0.04	0.22	0.27	0	0	0.12	0.01	0
CML338	4.47	0	5.94	2.66	0.04	0.48	4.15	7.19	1.83	1.98	4.27	1.59	0.08	3.28	0	0.35	3.85	3.59	0.91	1.42	0	0.09	0	0.65	0	3.4	0.02	0.13	0
SY1032	2.97	0	6.09	2	0.05	0.24	3.58	6.94	0.52	0	3.28	1	0	2.23	0.12	6.85	4.52	2.84	1.89	0.86	0	0.13	0	0	0	0	0.05	0	0
CML118	5.01	0	2.8	1.98	0.26	0	3.69	5.23	0.09	1.95	3.25	1.84	0	2.54	0	1.54	6.83	3.49	0.48	0.64	0	0.14	0.31	0.07	0	0.68	0.17	0	0
CML360	5.52	0	2.93	1.81	0.01	0.24	2.69	5.95	0.18	1.34	3.98	0.18	0	2.84	0	4.53	4.94	2.74	0.6	0.98	0	0.01	0	0.14	0	0.99	0	0.24	0
CML121	2.88	0	2.53	2.11	0.08	0	2.39	4.34	1.87	4.07	3.29	1.33	0.06	1.78	0.18	2.99	1.98	2.54	2.09	0.65	0	0.07	0.38	0.33	0	0	0.07	0.02	0
SY1035	4.68	0	3.79	1.93	0.09	0	2.08	5.85	0.11	2.93	3.69	1.63	0	1.17	0.23	5.42	4.69	2.62	1.07	0.52	0	0.12	0	0.08	0	0	0.08	0.14	0
CML361	2.98	0	4.83	1.28	0.71	0	3.73	3.93	1.86	3.68	3.01	0	0	2.96	0.11	4.24	4.01	2.99	0.82	0.74	0	0.01	0	0.37	0	1.92	0	0	0
CML122	4.06	0	0.49	2.45	0.07	0.5	2.49	7.51	1.12	3.73	3.73	0.61	0.02	0	0.27	3.37	3.32	3.12	1.11	1.15	0	0.23	0	0.16	0	0	0.03	0	0
SY1039	0.59	0	3.7	0.97	0.02	0.91	5.87	5.15	0.38	0	3.82	0	0.08	3.04	0.04	4.02	4.23	2.38	0.05	1.17	0	0	0.9	1.44	0	0	0.14	0	0
CML130	5.19	0	5.47	2.5	0.01	0.1	2.06	5.97	0.04	4.15	3.96	1.03	0	1.58	0.12	5.27	5.61	2.78	0.44	0.91	0.01	0.19	0.33	0.38	0	1.24	0.06	0	0
CML411	5.28	0	5.35	2.46	0.03	0	3.67	4.55	1.02	0	3.54	0.85	0.25	1.75	0	0.96	4.35	2.43	0.11	0.59	0	0.02	0.18	0.09	0	0	0.03	0.1	0
CML134	4.89	0	6.14	2.32	0.42	0	3.16	2.47	0.38	3.39	4.05	0.38	0	2.38	0.01	7.32	4.43	3.41	0.37	0.41	0	0	0.18	0.14	0	0	0.12	0.12	0
CML415	3.9	0	5.82	2.5	0.02	0.53	1.5	4.78	0.21	3.61	4.04	1.08	0	1.94	0	7.1	4.02	3.47	1.72	1.01	0	0.25	0.48	0	0	0	0.14	0.11	0
SY1052	4.65	0	4.79	1.67	0.41	0	2.4	5.17	0.22	0	2.93	0.15	0.02	0	0	6.88	3.82	2.1	0.4	0.41	0	0.09	0	0	0	0	0.08	0.04	0
CML139	5.07	0	0.44	2	0.02	0	4.04	5.29	1.87	2.79	4.04	0.24	0	0.39	0	0.95	4.3	3.11	1.12	0.82	0	0.12	0.34	0.39	0	0	0.1	0.06	0
CML422	2.33	0	0.58	2.61	0.19	1.38	3.63	4.62	2.6	1.48	3.47	1.65	0	2.45	0	1.36	2.29	3.22	2.35	0.54	0	0.02	0.76	0.25	0	0.92	0.03	0.11	0
CML162	1.98	0	5.72	2.32	0.03	3.47	2.27	6.61	2.75	1.4	3.75	1.2	0	0	0.38	1.46	3.04	3.95	4.61	0.48	0	0.06	0.38	0.36	0	0	0.11	0	0
SY1128	4.49	0	0.37	1.93	0.03	2.45	4.85	7.51	0.86	0	3.99	1.88	0	1.94	0.14	1.98	4.55	3.38	1.16	1.39	0	0.18	0.18	0	0	0	0.02	0.07	0
CML423	3.53	0	3.78	2.19	0.48	0.23	4.25	6.2	0.22	3.84	3.54	1.69	0	1.56	0	6.41	4.7	2.79	0.5	1.34	0	0.21	0.51	0.27	0	0	0.07	0	0
CML163	3.66	0	5.86	2.67	0.04	0.61	5.17	7.46	1.16	2.2	4.59	1.48	0	0	0	3.54	4.62	2.71	0.77	1.7	0	0	0.49	0.54	0.03	1.21	0.03	0.01	0
SY3073	4.2	0	4.09	2.04	0.02	0	3.66	4.1	0.61	4.01	3.27	0	0	1.73	0.1	1.75	6.99	2.06	1.36	0.45	0	0.01	0.32	0.19	0	0	0.22	0.01	0
CML426	2.17	0	2.41	2.35	0.24	0.25	2.67	3.82	1.66	4.17	3.49	0.01	0.03	0	0.1	1.1	1.91	2.86	1.61	1.01	0	0.4	0.1	0.12	0	0	0.17	0	0
CML165	3.66	0	3.93	2.8	0.58	1.43	3.19	5.34	3	2.75	4.49	1.47	0	1.64	0	1.63	4.56	3.42	2.15	0.61	0.01	0.13	0.35	0.58	0	0.62	0.02	0.12	0
TIAN77	3.56	0	5.21	2.59	0.16	0.43	2.9	5.25	0.23	2.48	3.57	0.18	0.08	1.63	0	5.96	2.76	3.14	0.55	1.3	0	0.09	0.25	0	0	2.12	0.19	0	0

CML431	3.51	0	3.76	2.73	0.82	2.29	5.11	6.05	1.02	4.75	4	2.95	0.09	2	0.29	0.85	2.97	3.5	0.73	0.45	0	0.23	0.15	0.37	0	0	0.34	0.05	0
CML169	4.76	0	0.05	1.76	0.25	0	3.86	6.89	0.03	0	2.89	1.35	0	2.74	0.04	0.49	6.07	2.61	0.23	0.95	0	0.09	0.05	0.07	0	0	0.07	0	0
TIE7922	4.93	0	0	2.25	0.66	0	4.47	7.19	1.55	2.47	3.75	1.34	0	1.91	0.14	4.96	4.87	3	0.92	2.1	0	0.02	0	0.24	0.02	0	0.04	0	0
CML170	5.2	0	5.79	2.2	0.04	1.31	5.11	3.78	1.98	4.15	3.77	1.69	0	2.98	0	2.11	6.6	3.01	0.47	1.47	0	0.34	0.28	0.73	0	0	0.09	0	0
CML432	3.61	0	0.19	2.17	0.07	2.54	4.68	4.13	0.03	1.87	3.91	1.38	0.09	0.59	0	5.37	4.74	3.45	0.6	1.26	0	0	0.41	0.67	0	1.27	0.03	0.03	0
CML171	4.1	0	6.08	2.55	0.02	4.21	4.98	4.25	2.84	4.59	4.22	2.78	0.04	3.33	0	5.5	4.86	2.99	1.34	1.07	0	0.08	0.93	0.22	0	2.81	0.04	0	0
CML433	4.47	0	0.6	2.45	0.12	0.91	4.26	3.67	0.2	3.61	3.71	0.48	0	3.17	0	4.44	4.57	3.16	1.21	1.25	0	0.15	0.18	0.11	0	0	0.04	0	0
CML172	2.58	0	5.99	2.94	0.94	0	3.77	4.3	0.64	0	4.03	2.58	0.41	2.78	0	4.87	4.69	3.99	0.79	1.17	0	0.11	0	0.19	0	1.95	0.03	0	0
TY1	4.13	0	3.55	2.58	0.02	0.06	4.4	6.6	0.73	4.86	4.47	1.88	0	1.75	0.05	8.47	4.31	3.14	1.62	1.06	0	0.05	0	0.06	0.04	0	0.08	0	0
CML454	3.84	0	5.84	2.68	0.05	1.03	5.36	8.02	2.6	0.01	4.41	1.78	0	4.25	0	1.1	4.5	4.04	0.96	2	0	0.08	0	0.39	0	3.22	0.03	0	0
CML189	5.32	0	1.37	2.96	0.06	0	4.21	9.02	2.87	2.57	4.13	0.65	0	3.58	0	1.07	7.06	3.5	0.5	0.55	0.03	0.02	0	0.13	0.07	0.88	0.03	0	0
TY11	3.83	0	2.57	2.1	0.04	0.31	2.65	5.23	1.32	3.89	3.75	0.66	0	1.11	0	5.41	2.04	2.93	0.7	0.15	0	0.04	0.7	0.08	0	0	0.12	0	0
CML191	4.87	0	5.61	2.47	0.11	0	3.96	5.97	0.04	0	3.57	0.04	0.02	2.31	0.23	0.86	7.26	3.4	0.3	0.94	0	0.14	0	0.39	0	0.99	0.1	0	0
TY2	1.74	0	3.2	1.89	0.03	0.46	0	2.59	0.8	3.32	4.03	1.34	0	0.6	0.02	4.89	4.67	3.02	1.52	0.44	0.01	0.04	0.04	0.18	0	0	0.08	0	0
CML192	5.16	0	0.42	2.69	0.92	2.22	3.89	3.09	0.29	0	3.62	2.18	0	3.4	0	7.43	4.77	2.92	0.79	1.34	0	0.75	0.34	0.07	0	0	0.07	0	0
Z2018F	4.77	0	5.57	1.62	0.19	0.44	2.94	3.96	1.69	4.38	3.76	0	0.14	2.2	0	1.24	5.38	3.18	0.4	1.17	0.01	0.02	0.27	0	0	0	0.17	0	0
CML20	2.74	0	2.97	2.24	0.06	0.13	3.06	4.25	1.39	2.67	3.6	1.3	0	0	0	0.85	6.65	3.38	0.97	0.9	0	0.07	0	0.26	0	0	0.07	0	0
TY3	3.43	0	2.52	2.3	0.03	0.15	2.23	4.92	0.01	3.93	3.43	1.14	0	1.52	0	5.71	4.46	3.01	0.39	0.53	0	0	0	0.13	0	0	0	0	0
CML290	4.53	0	5.25	2.47	0.03	0.03	3.66	2.91	0.81	2.86	3.85	0.02	0.08	0	0.81	0.79	3.98	3.29	0.78	0.61	0	0.1	0	0	0	0	0.06	0.18	0
ZAC546	3.21	0	5.77	2.33	0.25	0	1.74	3.23	0.27	0	3.74	0.76	0	0	0.13	5.43	4.45	2.91	0.13	0.28	0	0.06	0.28	0.27	0.06	0	0.18	0	0
TY4	4.74	0	2.48	2.17	0.03	1	4.27	6.87	2.96	0	4.08	2.41	0.03	1.63	0.12	8.1	5.23	2.76	0.5	1.87	0	0	0	0.18	0	0	0.04	0	0
CML298	2.8	0	4.35	2.17	0.17	1.84	4.24	5.05	0.42	0	4.68	1.99	0.17	3.38	0	4.51	4.8	2.75	0.52	0.86	0	0.12	1.49	0.96	0	1.14	0.09	0.62	0
ZB648	4.88	0	3.78	2.39	0.05	3.15	2.37	4.06	1.57	3.66	3.96	0.31	0	0.6	0.05	5.59	5.59	3.33	0	0.91	0	0.03	0.54	0.38	0	0	0.13	0	0
CML304	4.94	0	5.56	2.57	0.04	0.88	4.02	4.52	3.56	2.9	4.09	0.1	0.21	1.56	0	0.65	4.31	3.36	2.37	1.06	0.01	0.02	0	0.08	0	0	0.09	0.06	0
TY5	4.6	0	2.79	2.34	0.08	0	4.75	4.14	0.58	3.86	3.85	0.09	0.02	2.61	0.35	5.49	5.33	3.28	3.62	0.57	0	0.33	0	0.1	0	0	0.03	0	0
ZH68	2.28	0	0.78	2.66	0.38	0	1.98	9.18	1.76	4.63	4	1.76	0.24	4.08	0.24	1.91	4.77	3.01	2.36	1.97	0.01	0.22	0.1	0.35	0.68	1.7	0.19	0	0
CML31	4.31	0	5.36	1.82	0.11	0.02	2.56	4.38	2.96	3.64	3.79	2.18	0	3.07	0.03	2.11	4.8	3.24	2.85	0.4	0	0.08	0.23	0.42	0	0	0.06	0	0
TY6	3.46	0	2.97	2.35	0.14	0.54	2.73	6	2.61	0	3.6	0.14	0.06	1.65	0	4.67	4.7	2.65	1.69	0.12	0	0.04	0	0.08	0	0	0.26	0	0
CML32	4.77	0	0.48	2.05	0.05	0	4.68	5.47	2.49	1.92	3.98	0	0	0	0.11	0.85	1.91	2.96	0.75	0.4	0.01	0.01	0.52	0.04	0	0.77	0.04	0.26	0
ZHENG28	1.51	0	5.42	2.91	0.85	0.95	3.79	5.08	0.06	2.24	3.55	2.15	0	0.77	0	6.52	7.04	3.45	1.48	1.2	0	0.28	0.11	0.14	0.03	0	0.08	0	0
U8112	3.42	0	0.32	2.17	0.03	3.08	3.85	5.85	2.01	4.63	3.59	0.95	0	1.63	0.07	0.31	4.9	4.05	1.66	0.89	0	0.07	0.55	0	0.07	0	0.06	0	0
ZHENG29	3.4	0	6.34	2.86	0.03	0.36	5.51	5.9	0.3	2.77	4.13	1.66	0.13	1.67	0.02	1.72	2.88	3.38	2.45	0.9	0	0.15	0.11	0.48	0	1.02	0.25	0.1	0
CML323	3.95	0	5.98	2.12	0.14	2.03	3.96	5.78	0.36	0	3.37	1.62	0	2.56	0.03	1.25	2.68	4.12	2.07	1.12	0	0.16	0.11	0.55	0.01	1.94	0.19	0	0
W138	4.76	0	3.53	2.05	0.6	0.28	3.91	5.65	0.12	0	3.25	1.46	0	1.07	0.15	7.52	5.43	3.1	0.14	1.21	0	0.26	0.4	0.1	0.03	0	0.02	0	0
WH413	3.55	0	3.14	1.15	0.02	0.67	1.83	4.89	1.54	4.58	4.01	1.12	0	1.53	0	1.1	1.6	3.46	0.42	0.65	0	0.27	0.21	0.09	0	0	0.15	0.02	0
WU109	2.97	0	3.85	0.77	0.07	1.65	3.57	3.28	0.78	4.07	3.62	0.08	0	2.03	0	8.21	4.69	2.9	0.13	0.34	0	0	0.63	0.04	0	0	0.15	0	0
XI502	4.27	0	4.36	2.39	0.15	0	3.82	9.05	0.03	4.31	3.97	1.45	0	0.5	0	1.96	6.86	1.85	0.85	1.09	0	0.29	0.03	0.31	0	0	0.17	0	0
ZHENG30	2.54	0	4.86	1.95	0.53	0	2.6	4.56	0.08	1.67	3.39	0.54	0	1.89	0.07	3.4	6.02	3.04	1.14	0.55	0	0.19	0.21	0	0.03	1.84	0.12	0.01	0
XUN971	3.95	0	3.32	1.85	0.13	0.31	3.73	6.29	0.53	4.16	3.24	0	0	1.03	0.1	1.32	4.4	2.65	1.79	0.45	0	0.16	0.1	0.2	0	0	0.03	0	0
ZHENG32	3.7	0	0.2	1.12	0.11	0	1.5	4.43	0.88	4.09	3.52	0.08	0	0.34	0.31	1.43	3.73	2.54	0.71	0.61	0	0.19	0.44	0	0.4	0.33	0.2	0	0
XZ698	3.77	0	3.67	2.12	0.31	0	3.21	5.77	0.74	3.56	3.22	0.98	0	0.89	0	5.27	1.89	2.78	0.59	1.13	0	0.02	0	0.04	0	0	0.13	0	0
ZHENG35	4.5	0	4.78	2.43	0.05	0.18	3.49	6.47	0.04	3.47	3.44	0.97	0	1.72	0	1.7	2.06	3.06	1.3	0.86	0	0.2	0.23	0.46	0	0	0.13	0	0
YE478	3.47	0	1.46	2.36	0.78	0.48	5.03	5.83	5.26	2.16	3.71	1.86	0	4.26	0.06	0.34	5.27	4.21	2.33	1.98	0	1.02	1.07	0	0	0	0.03	0.11	0
YE515	3.63	0	4.96	2.39	0.31	0.8	3.48	4.36	0.12	2.64	3.28	1.79	0.03	0	0	0.66	2.64	3.57	1.76	0.73	0	0.27	0.28	0.16	0.13	0	0.02	0	0
ZHENG653	5.06	0	4.51	1.42	0.73	0	4.09	4.3	0.98	4.41	3.38	1.51	0	0	0	4.93	8.42	2.5	1.24	0.58	0	0.1	0.69	0.62	0	0	0	0	0
YE52106	4.17	0	0.18	1.95	0.27	0	3.91	4.46	0.12	3.26	3.41	0.43	0	1.71	0	4.15	6.8	3.84	1.45	0.44	0.03	0.06	0.26	0.21	0	0.7	0.04	0	0
ZHI41	4.51	0	5.71	0.41	0.52	0	3.05	1.51	2.05	0	3.64	0	0	0.14	0	5.14	3.8	2.99	0.77	0.29	0	0.01	0.3	0	0	0	0.04	0	0
YE8001	3.88	0	2.19	1.74	0.35	2.09	2.7	3.4	1.14	3.88	3.38	0.3	0.01	1.09	0	3.71	4.45	2.25	0.88	1.15	0	0.23	0.85	0	0	0	0.05	0.01	0
ZHONG69	2.72	0	0.24	2.41	0.45	2.37	4.99	5.56	3.35	0	3.76	1.64	0.04	1.9	0.56	6.92	4.75	4.07	0.58	1.28	0	0.12	0.84	0.1	0	3.11	0.01	0	0
YU374	4.54	0	5.65	2.02	0.11	0.27	4.48	5.62	0.33	1.45	3.47	1.54	0	0.81	0	4.72	7.06	3.32	0.2	0.73	0	0.16	0.32	0.22	0.01	0	0.06	0.05	0
ZONG31	4.35	0	5.85	2.35	0.06	0	4.45	5.51	1.11	4.31	3.27	0.01	0	1.09	0	4.01	6.69	3.03	0.31	0.44	0	0.13	0.28	0.2	0	0	0.07	0	0
ZZ01	4.89	0	3.47	2.27	0.34	1.98	2.92	6.02	0.34	3.54	3.26	0.11	0	1.39	0	5.52	2.89	2.82	1.55	0.05	0	0.1	0.28	0	0.24	0	0.13	0	0
ZZ03	0	0	3.82	1.72	0.12	0.73	2.85	0	0	0	3.29	0	0	0	0.64	10.77	6.88	2.47	0	0.49	0	0	0.29	0.23	0	0	0.43	0	0
